# Supplementary figures and images for: The recurring impact of storm disturbance on black sea bass (Centropristis striata) movement behaviors in the Mid-Atlantic Bight
Source: PLoS One. 2020 Dec 2;15(12):e0239919. doi: 10.1371/journal.pone.0239919 (PMC7710083; doi:10.1371/journal.pone.0239919)

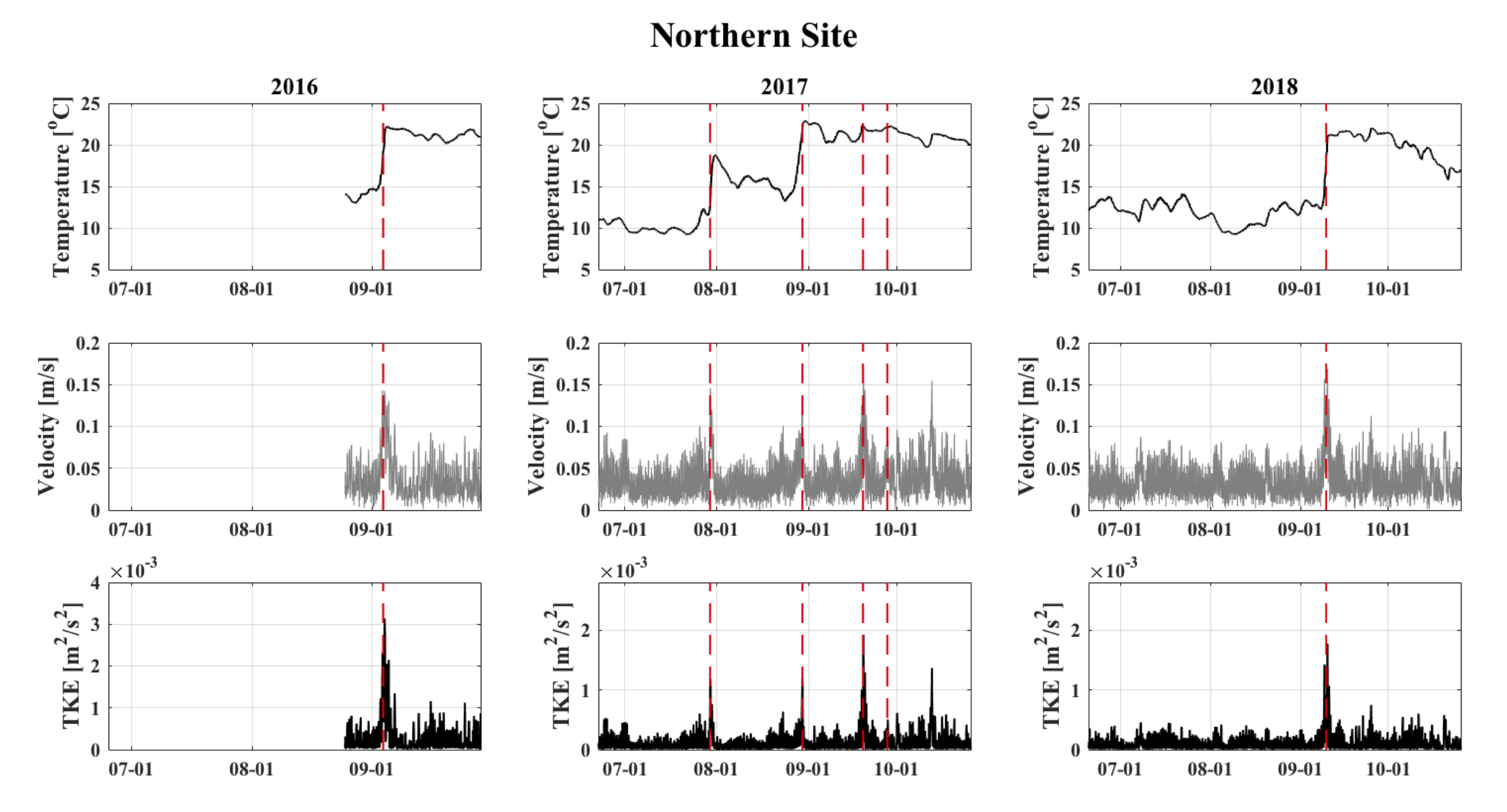

Supplement: S1 Fig — *Dashed red lines refer to modeled maximum wind speeds occurring during each of the six identified storm events (see Table 3). (TIF) [file pone.0239919.s001.tif]

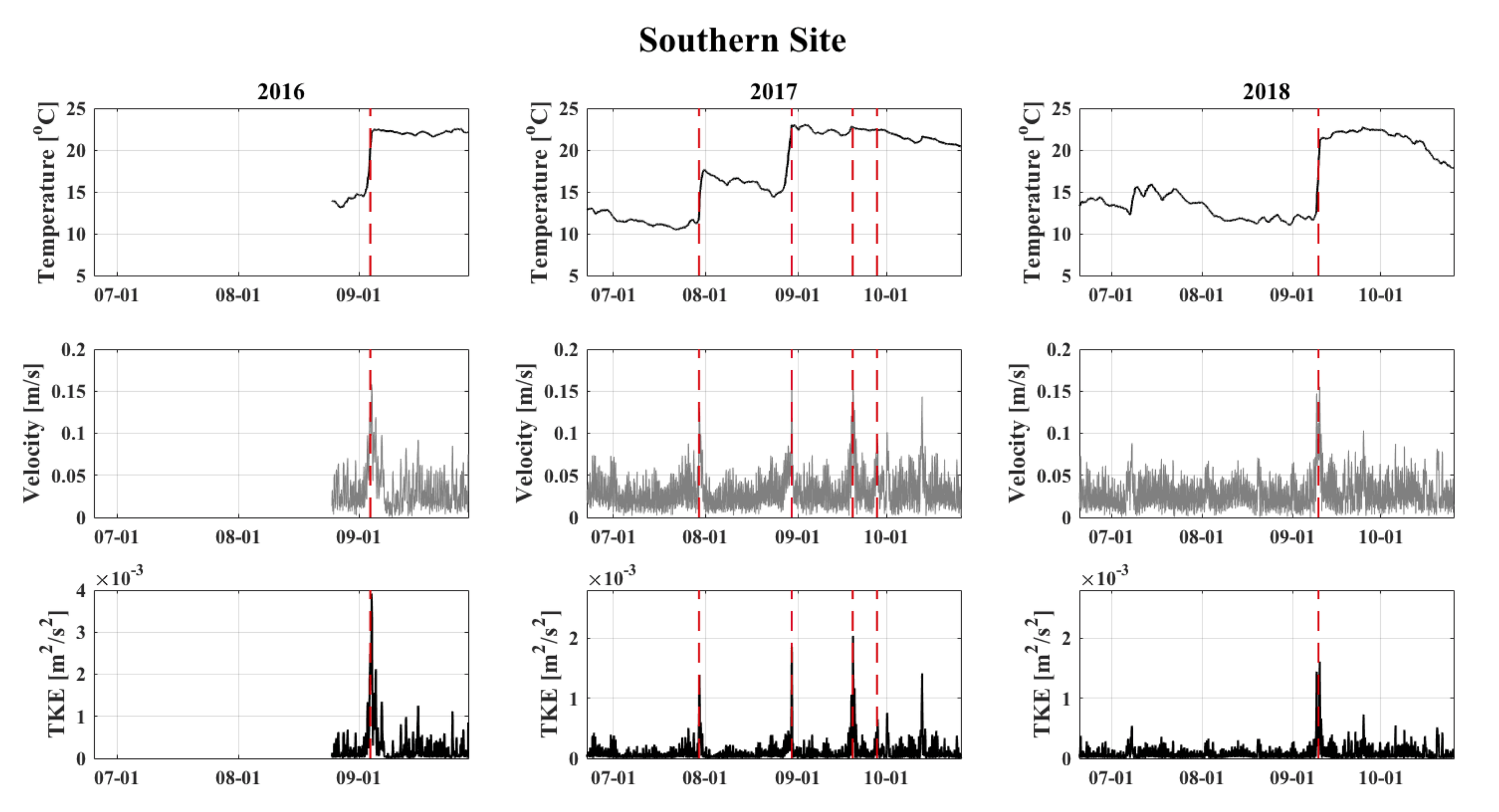

Supplement: S2 Fig — *Dashed red lines refer to modeled maximum wind speeds occurring during each of the six identified storm events (see Table 3). (TIF) [file pone.0239919.s002.tif]

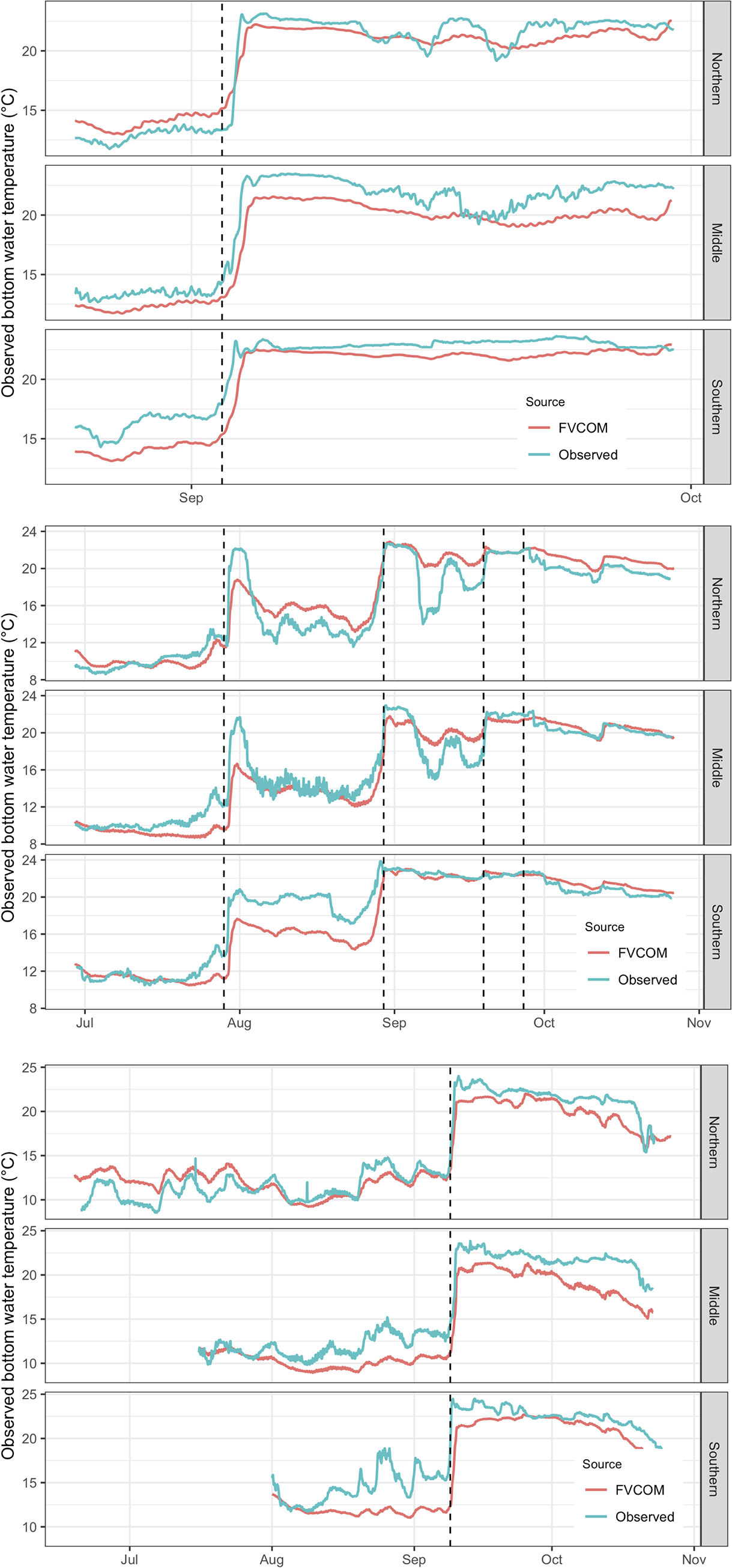

Supplement: S3 Fig — (a-c) Modeled and observed hourly bottom water temperature values across sites for 2016–2018, respectively. Vertical black dashed lines refer to maximum wind speed dates for identified storm events. (TIF) [file pone.0239919.s003.tif]

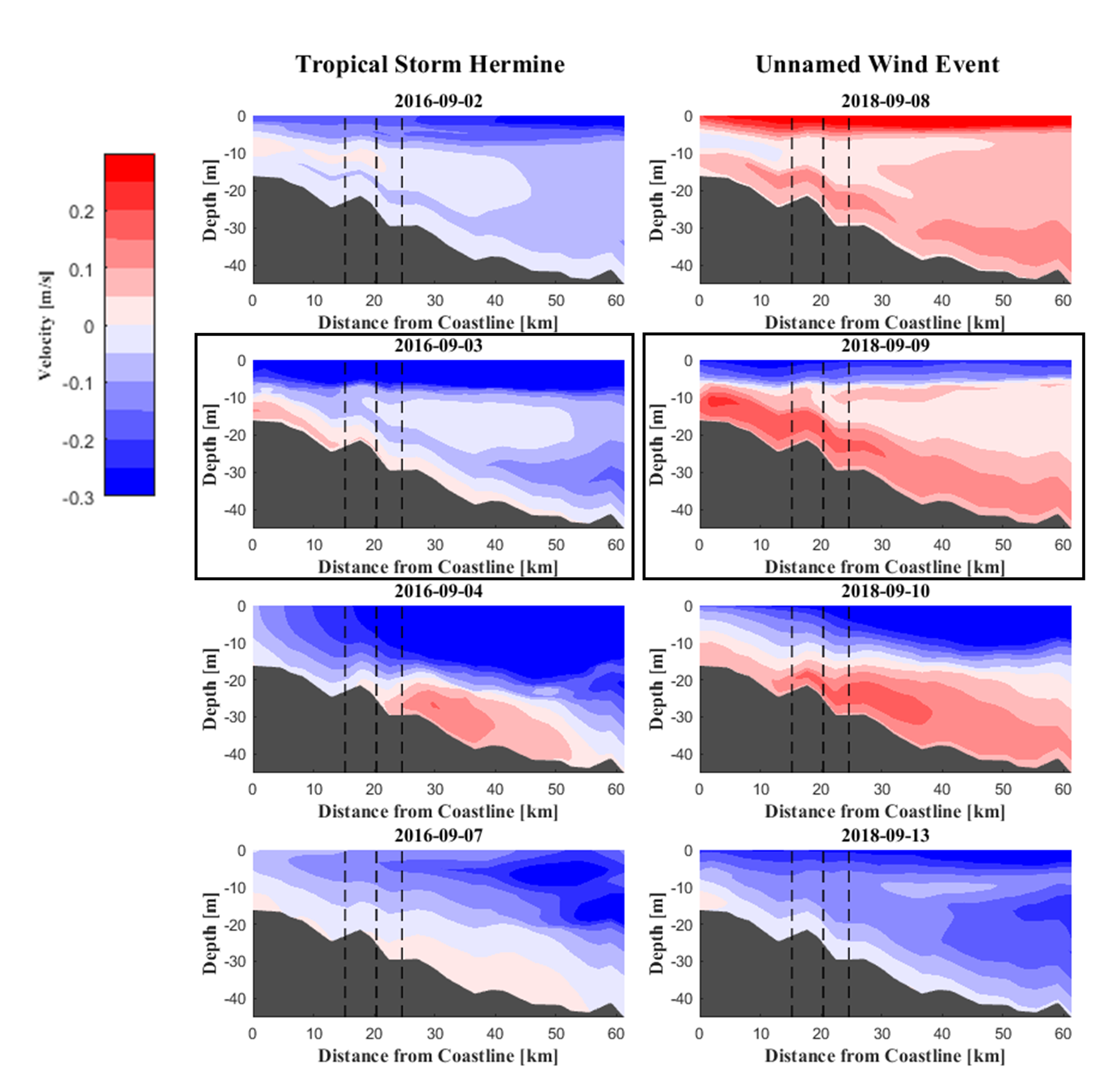

Supplement: S4 Fig — * Red colors refer to offshore current movement, and blue colors refer to inshore current movement. ** Vertical black dashed lines in each pane refer to the transmitter release locations central to each study site (Southern, Northern, and Middle, for both years in increasing depth and distance from coastline). *** Cross sections are taken along a transect spanning the Middle site, and depict predictions at 00:00 for each given day. **** Panes boxed in black refer to dates of maximum wind speed for the given storm event. (TIF) [file pone.0239919.s004.tif]

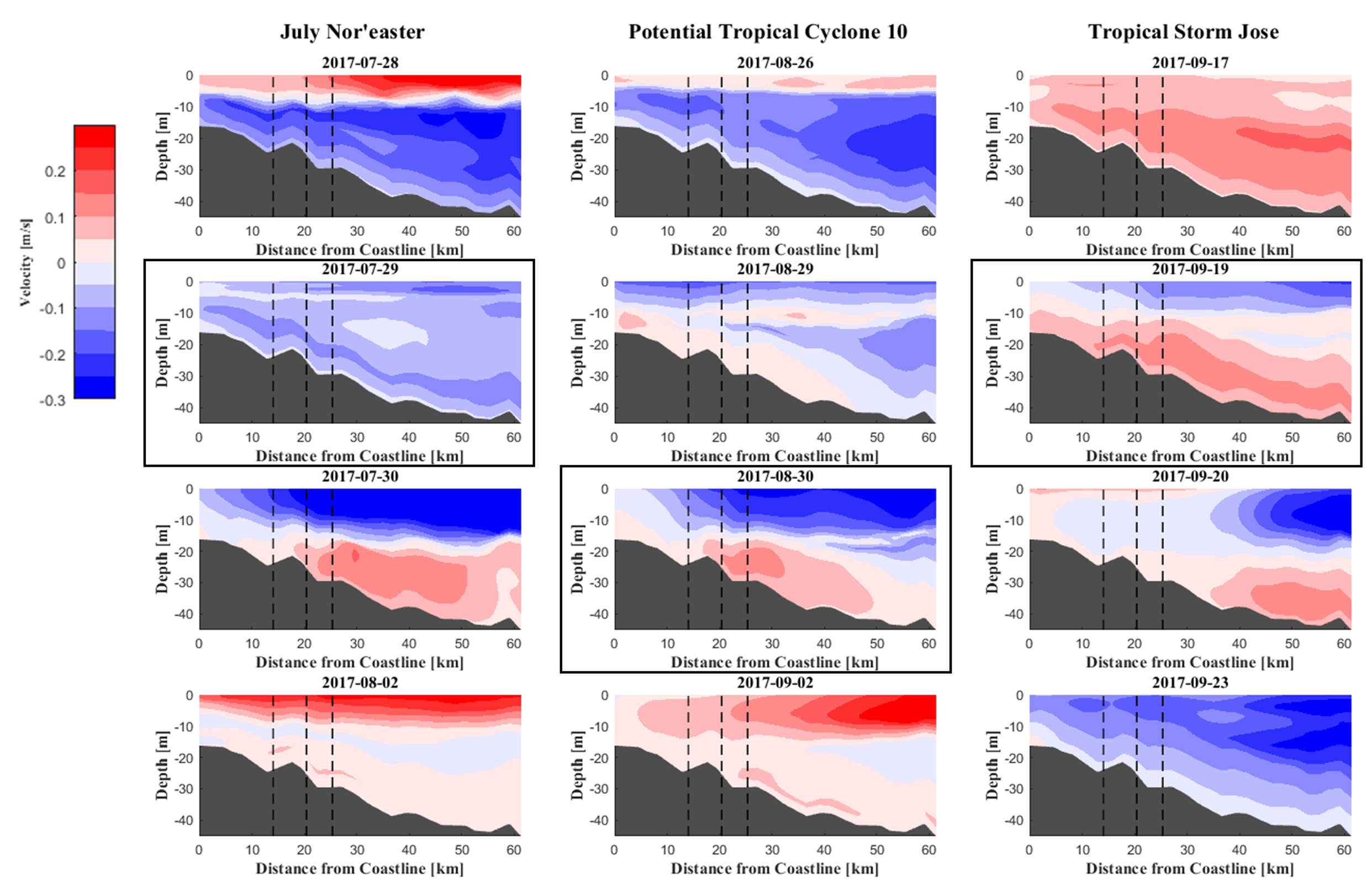

Supplement: S5 Fig — * Red colors refer to offshore current movement, and blue colors refer to inshore current movement. ** Vertical black dashed lines in each pane refer to the transmitter release locations central to each study site (Southern, Northern, and Middle, for both years in increasing depth and distance from coastline). *** Cross sections are taken along a transect spanning the Middle site, and depict predictions at 00:00 for each given day. **** Panes boxed in black refer to dates of maximum wind speed for the given storm event. (TIF) [file pone.0239919.s005.tif]

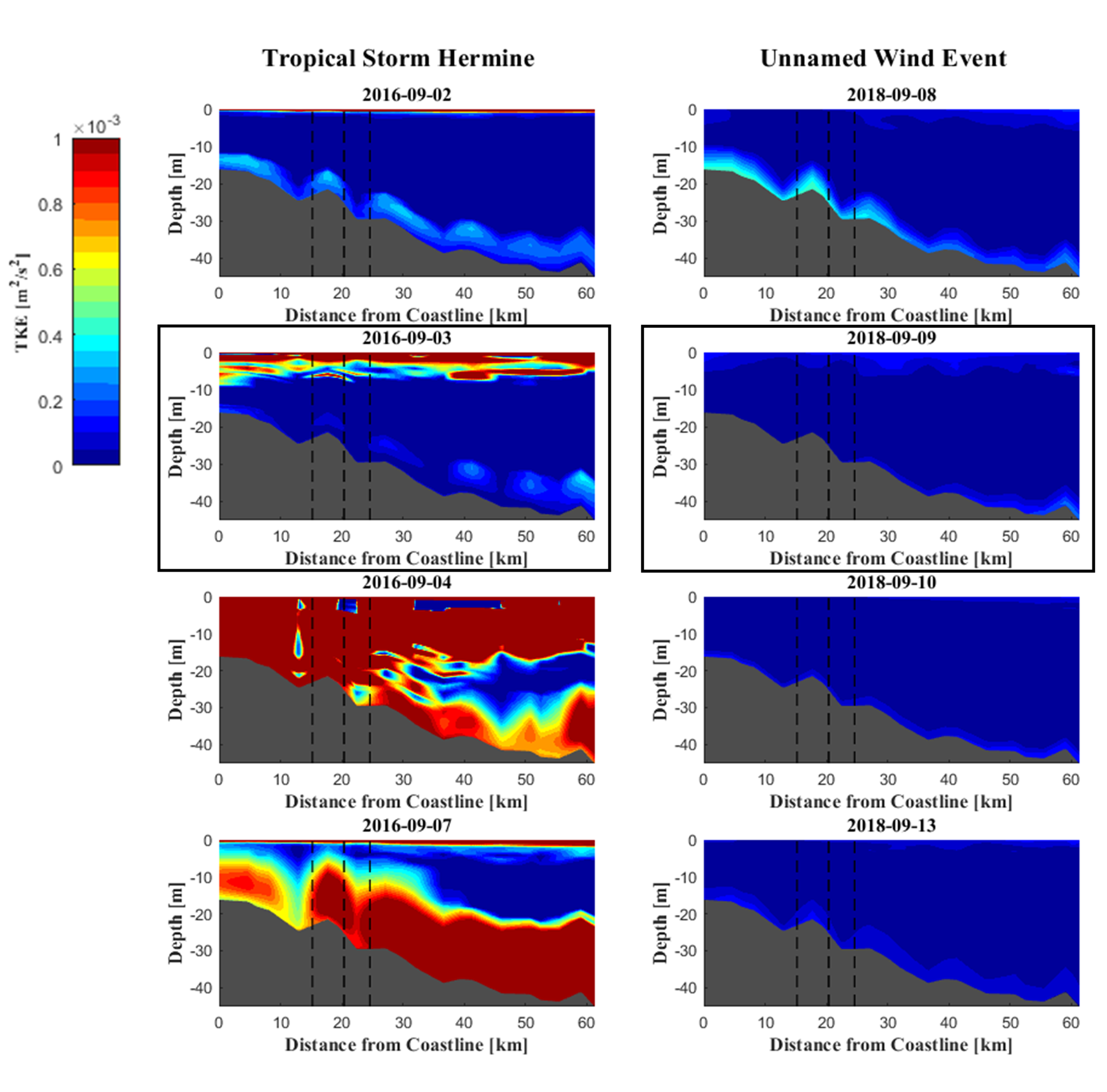

Supplement: S6 Fig — * Vertical black dashed lines in each pane refer to the transmitter release locations central to each study site (Southern, Northern, and Middle, for both years in increasing depth and distance from coastline). ** Cross sections are taken along a transect spanning the Middle site, and depict predictions at 00:00 for each given day. *** Panes boxed in black refer to dates of maximum wind speed for the given storm event. (TIF) [file pone.0239919.s006.tif]

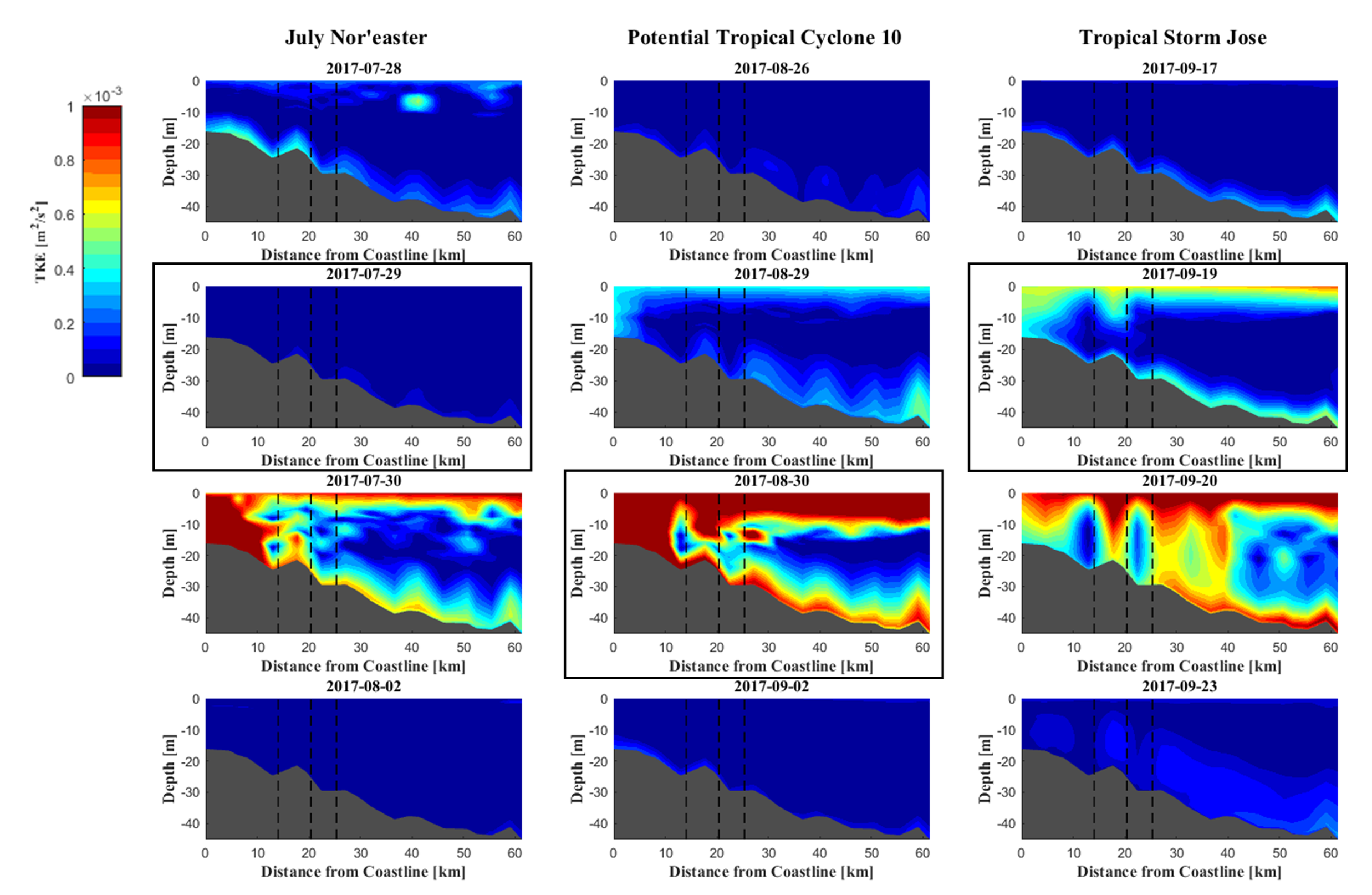

Supplement: S7 Fig — * Vertical black dashed lines in each pane refer to the transmitter release locations central to each study site (Southern, Northern, and Middle, for both years in increasing depth and distance from coastline). ** Cross sections are taken along a transect spanning the Middle site, and depict predictions at 00:00 for each given day. *** Panes boxed in black refer to dates of maximum wind speed for the given storm event. (TIF) [file pone.0239919.s007.tif]

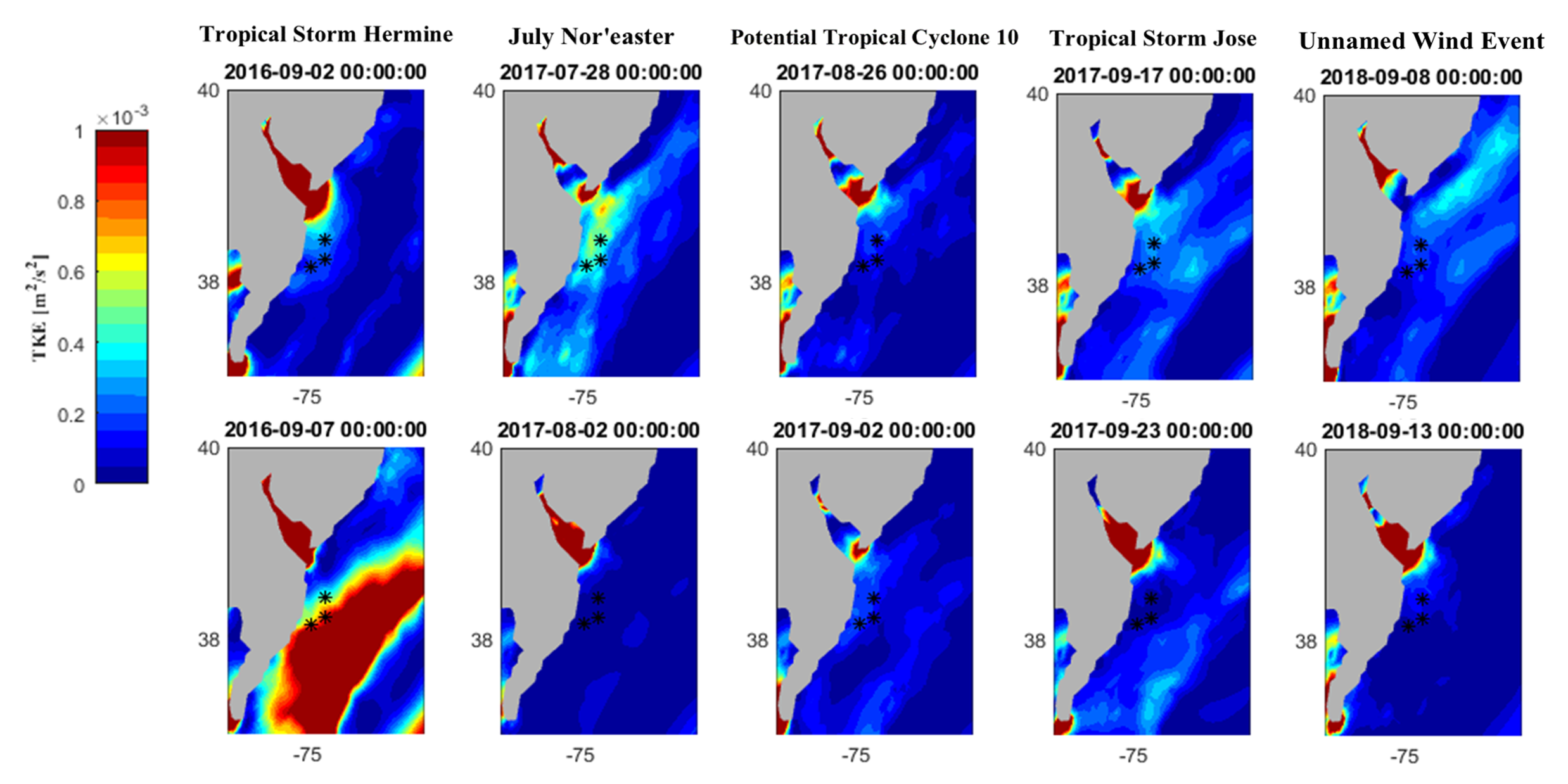

Supplement: S8 Fig — * Black asterisks refer to the location of transmitter release, central to each study site. (TIF) [file pone.0239919.s008.tif]

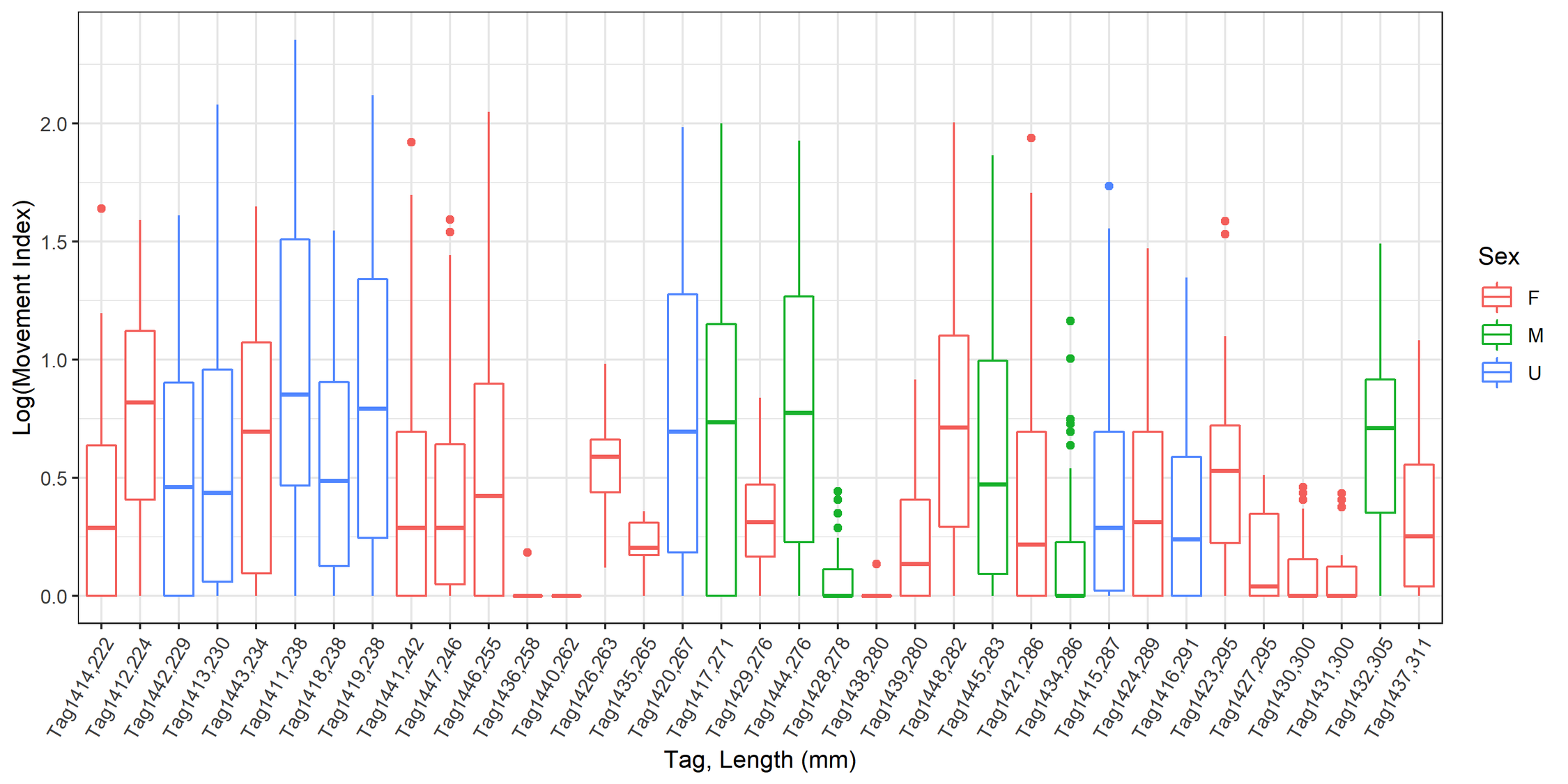

Supplement: S9 Fig — X-axis labels refer to the tag number, as well as the length of the individual (mm). (TIF) [file pone.0239919.s009.tif]
