# Supplementary material for: The recurring impact of storm disturbance on black sea bass (Centropristis striata) movement behaviors in the Mid-Atlantic Bight
Source: PLoS One. 2020 Dec 2;15(12):e0239919. doi: 10.1371/journal.pone.0239919 (PMC7710083; doi:10.1371/journal.pone.0239919)
Supplement: Attachment — Submitted filename: Review of Wiernicki et al.docx [file pone.0239919.s010.docx]

Review of Wiernicki et al. THE RECURRING ROLE OF STORM DISTURBANCE ON BLACK SEA BASS (CENTROPRISTIS STRIATA) MOVEMENT BEHAVIORS IN THE MID-ATLANTIC BIGHT. Submitted to PLoS ONE. Research Article

**General Comments/summary:**

Summary: The authors’ aim was to describe and analyze the impact of storms in the MAB in association with the physical breakdown of the cold pool and the effect on fish movement, specifically of black sea bass. This was achieved by using telemetry data and modeling the oceanography of the system using FVCOM. By using data from multiple years, the authors were able to show destratification of the MAB after significant storm events and evidence of changing movement behaviors of black sea bass after storms, which they likened to the rapid increase of bottom temperature and to a lesser degree of increased turbulence and current velocity. By chance, one of the study years (2017) included multiple storm events which allowed the authors to assess the effect of multiple storms on the system. Here they showed earlier storms did not have the same effect as later storms, but did gradually breakdown the cold pool.

Major comments: While this study, data, and results are important (and exciting!) for both understanding the impacts of storms on fish and for management of black sea bass, the manuscript needs to be improved substantially. Specifically, major edits are needed for:

1) Distinguishing the difference between low and high storm intensities: much of the literature is focused on the effects of hurricanes or large tropical storms. The MAB region receives many storms throughout the summer and fall months but only 6 storms are recorded in this study. While this is fine, the authors should distinguish between background levels of storms vs. the more severe storms focused on in this study;
2) The physiological effects of storms on fish: authors used minimal evidence to argue negative effects of storms and the links between stressors and physiology are not well developed here. Are the authors just arguing that warmer bottom temperatures cause black sea bass to reduce movement to conserve energy? Much of the issues related to this point can be found in the specific comments;
3) Placing the study into context with variability of fall overturn in this region: these storms are important for fall overturn and their variability likely leads to variability in fall overturn. This is important and should be developed more;
4) Distinguishing between the behavioral responses of decreased movement vs. evacuation: these are very different responses, having varying impacts on the population and should be discussed more. Especially as much of the literature used in this manuscript only focuses on evacuations of fish;
5) Distinguishing which months the authors specify as summer and fall months which likely should reference seasons based on the literature focused on fall overturn in the MAB. The authors refer to many of the storms as summer storms when they are actually occurring during the beginning of fall overturn. This is important because stratification during summer is very strong and summer storms do not typically break up stratification. Fall storms will break down stratification and is already known to be a time of significant change in the system and well documented movements of fish species;
6) Explaining if there were any occurrences of fish returning after a storm event. This has been seen in some papers and would be interesting if this happened to black sea bass, especially during the earlier storms of 2017. If there is no evidence the fish returned or fish returns were not detected, that is fine but should be explained;
7) The text overall should be edited to improve clarity and readability. Some cases of this are specified in my specific comments and edits.

**Specific comments/edits – Text, Tables, Figures:**

***Title***

The phrase “recurring role of storm disturbance” in the title is ambiguous. Are the authors referring to recurring storms and their impact or that there is continued evidence of impacts of storms on black sea bass?

***Abstract***

Specific (bold = insert):

Line 25: Summer storms do not cause rapid destratification in the MAB. Fall storms yes, but not summer. Please revisit classification of what is considered a summer or fall storm. I have multiple comments about this in the rest of the specific comments.

Line 28: “model ubiquitous demersal” – while black sea bass relatively stationary behavior makes them ideal for telemetry studies, it is not clear why the reader should care about depressed movements of an already stationary fish. Perhaps rephrase to emphasize “model” organism relates to their use in tagging studies but not as much on the potential impact of storms.

Line 33: is this 8-15 black sea bass per year or over the entire 2016-2018 time frame? Please specify.

Line 33: “at each of three reef sites” – this is confusing as the authors have not introduced the three reef sites yet in the abstract. I suggest rephrasing this statement.

Lines 34-35: “…activity levels **and** reef departures **of black sea bass,** and fluctuations in **modeled** temperature …”

Lines 36-37: How do the authors reconcile differing behaviors of low activity vs. evacuation? Is this an artifact of the methodology or truly binary responses to the storm events? How do the authors know if late season departures are not associated with offshore migration?

Lines 40-42: The conclusion of the abstract feels like a stretch. Where are anthropogenic impacts measured in this study? What are the anthropogenic impacts (i.e. fishing? Climate change? Pollution?). Perhaps a stronger conclusion is to place these results in context with climate change and the predicted increase of storm frequency and intensity along the MAB in the future.

***Introduction***

General: I would advise the introduction to be reorganized so that the reader is not switching back and forth between reading about storm impacts and the MAB. Perhaps addressing the storm disturbance and impacts on fish first and then introducing the MAB system, the effects of storms on the MAB and potential impacts on black sea bass would flow better. Also, the introduction is a great place to distinguish which months of the year are classified as summer and fall. Finally, in the description of the MAB, the variability of the fall overturn should be explained, perhaps also in reference to changing phenology of the system, so that the storm events measured can be placed into context of interannual differences in the MAB.

Specific (bold = insert):

Line 50: “less physical structure” – This is intriguing but what might be the potential difference or concern between a region with varying degrees of physical structure?; “subject to hurricane-forcing” – this statement feels out of place because some of the other regions referenced above are not subject to hurricane forcing. Is this insinuating that we are only concerned about regions with hurricane-forcing and those with lesser storms are not as important? *This is an area where the authors could develop their ideas further. For example, what makes the MAB unique compared to these other systems is the cold pool and the ability for it to breakdown during storms. Placing this into context with the other systems referenced, especially in relation to rapid temperature changes, would provide a stronger argument to why the MAB system is important to study.*

Line 53: “…Cape Hatteras, **North Carolina,**…”

Line 58: “…MAB is also vulnerable to storm-driven **temperature** disturbances …”

Line 59: Remove “overlapping”.

Line 61: “…at **the** Nantucket Shoals.”

Line 64: References 13-16 mostly focus on fall overturn. This is fine but the seasonality of the cold pool destruction should be clearly stated. If there is more evidence of summer storms impacting the cold pool (besides this study), I would also add those and again be clear about what part of the year this is occurring.

Line 66: How common are nor’easters in the summer?

Line 68: “summer storms” – again, this should be clarified in terms of what months “summer” refers to

Line 69: “rapid partial destratification” – partial vs. full destratification should be explained

Line 70: The values of temperature change are based on a hurricane event. This should be clarified as an extreme event in the system.

Lines 70-71: Refs 18 is focused on juvenile black sea bass not adults and also gradually increased temperatures after acclimation; and refs 19-20 are on temperature tolerance not so much acclimation and very acute exposures. The impact of rapid temperature change on fish physiology is extremely important but this section needs work. Can the authors find studies of acute vs chronic exposure of increasing temperature? Studies on heat shock proteins? Also, there needs to be a link between how the physiological stress of increased temperature will affect fish behavior (i.e. movement). Is there a threshold where a fish cannot move and will not evacuate? Did any of the other papers on fish responses to storms report physiological stress? Here is also where there should be some distinguishing between behaviors of avoidance or mitigation? For example, are the fish leaving the system before the storm or compensating during and after?

Lines 73-75: I am intrigued about potential issues related to changes in current velocity, TKE and noise but the authors do not provide further evidence that these are stressors to fish, and as such do not provide a potential hypothesis as to how these physical effects from storms could affect fish.

Lines 75-76: “Because several storm systems …destratification each year” – this statement is awkward, rewrite.

Line 77: “disturbance regime” – this seems to be an important concept… the authors should explain this further and show an example of other disturbance regimes to provide evidence to why this should be unique on the MAB

Line 87: Rewrite as - “Thus, the literature supports that **severe** storms can disrupt demersal fish communities”. – along these lines, the examples from the literature only provide evidence of fish evacuations not reduced movement behavior…is there evidence for this in other fish species? It would be useful to provide a variety of responses of fish to storms especially because the authors found depressed movement. If this study is the first to show depressed movement, then this should be explained later in the discussion.

Line 87-89: “Still, the concept … been fully explored” – this statement needs to be developed more. Do all studies above represent singular extreme events? Why is it important to study if storms occur each year in comparison to the other studies?

Line 92: “range behaviors” – this is awkward, please rephrase.

Lines 92-94: Re-write as “…centered on artificial and natural structure [25] and are mostly sedentary and reef-associated [26-29], making them amenable to biotelemetry studies on their movement behaviors.”

Line 95: “…occur **inshore** throughout the **spring**, summer and early fall months,…”

Lines 94-97: “Black sea bass and … late October [26,27,30]” – this sentence is awkward, please rewrite this sentence

Lines 96-100: So here the authors explain black sea bass movements in relation to their fall migration…so are the storms analyzed in this paper not fall storms? Again, clarifying summer and fall months early on in the paper should help reduce confusion surrounding this issue.

Lines 99-100: The hypothesis from ref 17 ends the paragraph without any additional follow up from the authors. Is this also hypothesized for this paper? If not, how are the hypotheses and results of this paper placed into context with this hypothesis?

Line 101: Replace “is” with “was”

Line 103: Remove “More specifically” and capitalize “We..”

Lines 103-108: Are there any hypotheses related to differences in movement behavior (i.e. depressed movement vs. evacuation)?

***Materials & Methods***

General: The authors did a good job explaining the tagging, telemetry, ocean modelling and data analysis. I had a couple of concerns with the statistical analyses. There is no discussion about sample size in the GAMLSS. For example, there was a positive influence of being male on movement behavior (in results section) yet there are only 7 males included in this analysis. There should be some test or analysis to show that the sample sizes used in this model are adequate. Also, for the daily movement index, how does the data maintain independence if the daily movement can include movement of one fish over multiple days? If this is incorrect, then please clarify this in the methods. Besides overall text editing (the authors switch between present and past tense), the rest of my comments are specific, see below.

Specific (bold = insert):

Line 115: Rewrite: “This project included three study reef sites located …”

Line 116: “…Ocean City, Maryland, **USA**, …”

Line 118: “were exposed to” – rephrase this statement; exposed to sinuates some experimental manipulation not that these sites regularly are part of the cold pool

Line 147: Was the tank a flow-through tank? If so, this should be mentioned. If not, were there airstones?

Line 151: After anesthetizing, the authors should include weighing, measuring and sexing the fish. How the authors sexed the fish is very important because they do not explain how they arrived to Unknown statuses for some of the fish in the study and this is important, especially for black sea bass.

Lines 153-154: “…was made cranial to the vent, and lateral to the midline.” This phrase is confusing, please rewrite.

Lines 157-159: Did tagging the fish not help reduce some barotrauma? If the fish were fully recovered in the tanks post tagging, then how did some of them need help equalizing in the water? Were the fish vented at all? Did the fish get external tags so that if they were accidentally caught fishermen could notify the researchers?

Line 166: Change “Mid-Atlantic Bight” to MAB

Lines 170-171: Are there citations for the ROMS ESPreSSO model?

Lines 177-178: The authors mention potential stress from current velocity but there is not cited evidence of this for fish nor is this idea developed in the introduction. Also what do the authors mean by “station-keeping”?

Line 185: “Delmarva” – this needs to be explained; most readers not from the US East Coast will not know what Delmarva means

Line 187: How exactly were the model results evaluated with the observed bottom water temperature? See my comments for figure SI3a-c.

Line 191: How and where did the authors measure peak wind speed?

Line 195: Change “evens” to “events”.

Line 193: How was the threshold of >5m/s chosen? This seems to have filtered out less severe summer and fall storms that occur in the region. In my opinion this is fine, but the presence of only severe storms in the analysis should be explained here and in the discussion.

Lines 216-217: “This approach allows discrimination … caused by storms.” – How is this interpreted to distinguish between fish leaving and coming back vs. false detections of a fish not actually leaving?

Lines 233-235: Did the authors only run one model of 2017? If so, why did they not run models for 2016 and 2018 and remove the ANSD term? This sentence is a bit ambiguous to the reader.

Line 259: How were the different models constructed, considering the authors picked the best fit model based on AIC scores?

***Results***

General: Once the determination of summer and fall is better described, the authors should edit the respective statements accordingly. This applies to the entire results section.

Specific (bold = insert):

Line 280: Rephrase to clarify that the storms occurred between the measured time period of June to October (because the storms did not occur in June or October)

Lines 282-286: The storms in September to me are early fall storms and the only true summer storm was the one at the very end of July; the August storm is also pretty late for summer. So the storms assessed are really more focused on late summer-fall storms and not “summer storms”. But if during editing this is clarified prior, then please disregard this comment.

Line 301: The relatively stable range of 12.5-16.9°C is still a significant change in bottom temperature (and significant for biology).

Line 305: What values are in the parentheses? Mean and SD? If so, this should be clarified.

Line 306: Rewrite – “…a destratification event *at the end of* July,…”

Line 314: “stronger storms and more moderate storms”. This seems subjective; was there a way to classify storms as “strong” vs. “moderate”?

Line 322: In looking at Figure 3, the winds appear to be northeasterly not northwesterly.

Line 381: Replace “all” with “each”

Line 384: Replace “lowered” with “decreased”

Line 385: Replace “than during” with “compared to”

Line 385: What pairwise comparisons were significant? Is this between site? Or between years?

Lines 385-386: I am confused why the results for 2017 are only in reference to PCT10. Did the authors group together data before and after PCT10? What about the other storm systems? Again, for the Tukey contrasts, is this across sites? It would be nice to know which sites were significantly different from each other.

Lines 394-396: Does this mean that when there are more frequent storms there is more evacuation from the shallow site compared to when there are less frequent storms?

Lines 396-398: I am confused. The authors state that the instantaneous loss rates at the Middle site were the highest during all years except for 2017 and mention that this occurred when the Middle and Northern evacuation rates were similar, but really this was when the Southern site evacuation rate was higher (i.e. Middle and Northern were similar but NOT higher than Southern).

Lines 397-398: For readers unfamiliar with telemetry studies, it would be nice to put into context the difference in instantaneous loss rates across the years. There are only slight increases in rates; is this still significant? Or does it mean that there is not much interannual variability in evacuation rates all things considered?

Line 437: Is this from the results of the best fit model? If the authors went through model selection it would be nice to have a clear indication of which model performed the best

Line 439: So along the lines of my comment above, the best fit model still included the non-significant ANSD term?

Lines 442-443: Remove “although it did not directly relate to storm influence on movement”. This is not quite known and it is obvious there is other biological information included in the model.

Line 445: If there might be an interaction between sex and length, why was this not included as an interaction? I agree that there likely is an effect of sex on length. If this is not to be used as an interaction, it would be worthwhile for the authors to provide a preliminary test showing there is or is not a relationship between sex and length.

Line 449: “…entire sample.” – what do the authors mean by “sample”? I am confused by this statement

Line 460: Remove “the” after exhibiting.

***Discussion***

General: What seems to be an important and exciting result is that it appears the evacuation of black sea bass really only occurs in full when the storms are in the fall (September months) and this is not affected by prior storms in the late summer (in 2017). The author’s mention the hypothesis that Secor et al. pose (fall storms are the trigger for offshore migration) and these results in my opinion add supporting evidence of this. However, this result is never fully developed nor placed into context with the hypothesis above. I suggest revisiting it. Also, the authors need to place their results in context with other fish studies in the end to compare how these results may be different or similar to other study systems. Finally, the last paragraph on offshore wind energy seemed very out of place, and definitely not a good conclusion to the paper. There are many more important aspects that the results of this research can be placed into context of (i.e. climate change and increasing storm frequency, shifting fall phenology in the MAB, etc.). The effect of noise, electromagnetic fields, and prey fields was never analyzed in this paper and the relation to storms and development of offshore wind farms seems out of place and a stretch to make. If this is actually a main theme of the paper, then I highly suggest the authors edit and introduce the impacts of wind energy in the introduction when describing the MAB system. But my suggestion is to not include this information. Wind energy is definitely an important and timely study area, but this study did not really address wind energy in the analyses and the results of this study are important enough to stand without tying them into wind energy.

Specific (bold = insert):

Line 472: “…changes **in** temperature,…”

Line 483: “Early summer storms” – Not trying to beat a dead horse, but a storm occurring at the end of July does not seem “early” to me.

Line 494-495: It seems misleading to mention that evacuations are an extreme response yet they happened every year in the study without including information that these are also occurring during the time that we expect to see black sea bass start to move offshore.

Line 504-506: Does this sentence thus indicate that the influence of Maria on water temperature did not instigate as rapid of a temperature change as the other storms? If so, this should be clarified.

Lines 516-518 and 521-522: These seem to contradict each other. Do these sentences indicate that the Southern site temperature varies more in frequency than in magnitude when compared to the Middle and Northern sites? If so, please clarify.

Lines 525-526: “The lowest rates of transmitter loss and the lowest number of evacuations occurred at the Southern site for all years” – But the authors found higher rates of instantaneous loss rates in the Southern Site in 2017…? I am confused.

Line 530: “…relatively shallower Northern” – on the previous page the authors mention that the Northern site is deeper and similar to the Middle site. Please correct and clarify this.

Lines 532-540: This paragraph is a good section to include other studies and place the results of this study into context with other fish responses to storms.

Lines 538-539: If the results from Fabrizio et al do not have the same results as the results for this study, what potential reasons can explain this? Perhaps this can shed more insight into the results found in this study (i.e. comparing the storm system of that study to the ones in this study).

Line 561: Refs 55-57 discuss reduced movement in relation to predatory prey experiments and indirect effects found in communities so I am confused how this can be related to reduced movement from storm impacts (these are very different mechanisms)

Line 565: Ref 27 describes the biology of the South Atlantic stock of black sea bass. Please reference a paper for the northern stock of black sea bass, the species that was used in this study. Also, the September-October is towards the end of the spawning season in this region so I don’t quite know how much of an effect there would be. To me, a more important and concerning aspect is the effect of storm systems on recruitment and the ability for recently spawned gametes and larval fish to ingress during these events. Also occurring at this time is the potential for black sea bass to change sex and this should at least be addressed here as the authors found important differences between the sexes and movement behavior.

Line 573: “…induce range shifts…” – what do the authors mean by this? In what way do they expect range shifts to occur (i.e. latitudinally? Cross shelf?)

Line 575-588: Refer to my comment in the general comment about this paragraph.

***References***

Some of the references were not inputted correctly (e.g. Ref 16, 32). Please review all references and make sure the citations are accurate.

***Tables***

Table 3: I suggest reformatting so that the respective data for each storm is easier to follow (i.e. the “Name” column looks like a string of words in a column, it took me a bit to figure out which events related to which values in the rest of the table).

***Figures***

General: There are many figures that do not have the “°” before C for temperature. Please incorporate this where it is missing.

Figure 1: Why is the buoy indicated in the map but the data from it never used? Or if the data from it was used, then this was not clear in the manuscript. Also, there should be an inset map showing this region across the broader US East Coast for readers unfamiliar with this region.

Figure 3: There should be a note about the different x-axes. I suggest instead replotting 2016 to be on the time scale as 2017 and 2018 and just begin the data for where its available.

Figure 4: Similar comment to above, the x-axes should all be the same. Also the difference in the y-axes should be mentioned in the figure caption. Also the figure caption only includes a description about temperature but needs to also include it for velocity and TKE.

Figure 5: I suggest placing a box around the panel for each event that indicates when the peak winds for the storm occur. This will make it easier for the reader to view the results (which look really nice!) without flipping back and forth between the table with the dates of the storms.

Figure 6: If the authors are including three events from 2017, why not include all four? It is a bit confusing why one is not included.

Figure 8: I suggest replotting to have all graphs have the same y-axis so they can be compared across the years. Also, the date ranges are confusing for the 2017 panel. It is unclear which dates (and associated box plots) indicate before or after a storm. Also, some of the years have a lot of variance. Is there a reason for this?

Figure 9: The red circles and the mean behind red dashed and solid lines are not included in the figure caption. Please include this. Also, where is the data for the Northern Site 2018? I realize the model did not converge but it did not for other regions and years yet they have all the data plotted.

SI1-2: Same comments as for Figure 4

SI3a-c: It appears there is some model bias with FVCOM on average underestimating the observed temperatures yet this is never addressed in the manuscript. How was this dealt with? In some cases the bias was almost on the order of 5°C which is very significant for fish (e.g. Southern Site 2017).
