# Supplementary material for: The recurring impact of storm disturbance on black sea bass (Centropristis striata) movement behaviors in the Mid-Atlantic Bight
Source: PLoS One. 2020 Dec 2;15(12):e0239919. doi: 10.1371/journal.pone.0239919 (PMC7710083; doi:10.1371/journal.pone.0239919)
Supplement: Attachment — Submitted filename: Response to Reviewers.docx [file pone.0239919.s011.docx]

Dear Dr. Magar:

Thank you for your very thorough review. We know that this takes a great deal of time and care on your part to ours and the manuscript’s benefit and improvement. We have addressed all general and specific comments below, referencing line numbers for more substantive changes in the clean/marked copy.

Sincerely Yours,

Caroline Wiernicki, NOAA Knauss Fellow

cc:	M O’Brien

F Zhang

V Lyubchich

M Li

D Secor

Review of Wiernicki et al. THE RECURRING IMPACT OF STORM DISTURBANCE ON BLACK SEA BASS (CENTROPRISTIS STRIATA) MOVEMENT BEHAVIORS IN THE MID-ATLANTIC BIGHT. Submitted to PLoS ONE. Research Article

**General Comments/summary:**

Summary: The authors’ aim was to describe and analyze the impact of storms in the MAB in association with the physical breakdown of the cold pool and the effect on fish movement, specifically of black sea bass. This was achieved by using telemetry data and modeling the oceanography of the system using FVCOM. By using data from multiple years, the authors were able to show destratification of the MAB after significant storm events and evidence of changing movement behaviors of black sea bass after storms, which they likened to the rapid increase of bottom temperature and to a lesser degree of increased turbulence and current velocity. By chance, one of the study years (2017) included multiple storm events which allowed the authors to assess the effect of multiple storms on the system. Here they showed earlier storms did not have the same effect as later storms, but did gradually breakdown the cold pool.

Thanks for this very succinct summary of the paper.

Major comments: While this study, data, and results are important (and exciting!) for both understanding the impacts of storms on fish and for management of black sea bass, the manuscript needs to be improved substantially. Specifically, major edits are needed for:

1) Distinguishing the difference between low and high storm intensities: much of the literature is focused on the effects of hurricanes or large tropical storms. The MAB region receives many storms throughout the summer and fall months but only 6 storms are recorded in this study. While this is fine, the authors should distinguish between background levels of storms vs. the more severe storms focused on in this study;

Thanks for this request for greater specificity. We have used the Beaufort Wind Scale and more careful reporting of wind speeds to distinguish higher intensity storm and wind events throughout the paper. Of course, it’s not just about wind speed – pre-existing ocean state, direction, and other aspects will impact the intensity of a given storm, but we do agree some criteria are needed here.

2) The physiological effects of storms on fish: authors used minimal evidence to argue negative effects of storms and the links between stressors and physiology are not well developed here. Are the authors just arguing that warmer bottom temperatures cause black sea bass to reduce movement to conserve energy? Much of the issues related to this point can be found in the specific comments;

A valid point. We have addressed this below, but were aided by discovering a recent paper specific to black sea bass that allowed us to estimate reduced aerobic scope associated with a change from 12 to 24 C, which was fairly typical for destratification events. Other more general literature has also been added on likely physiological effects.

3) Placing the study into context with variability of fall overturn in this region: these storms are important for fall overturn and their variability likely leads to variability in fall overturn. This is important and should be developed more;

We agree that the role of storms in driving fall overturn in this region is important. However, while the three-year stimulation discussed in our study has shed some light on how storms affect the fall transition process in the water column from highly stratified to well mixed, we believe it is far from sufficient to explain the interannual variability of fall overturn in this region. For inter-annual variability studies, at least a decade-long simulation is typically needed to provide a reliable assessment of how different storm characteristics affect the timing of fall overturn. We believe this is beyond the scope of this paper.

4) Distinguishing between the behavioral responses of decreased movement vs. evacuation: these are very different responses, having varying impacts on the population and should be discussed more. Especially as much of the literature used in this manuscript only focuses on evacuations of fish;

We have sought to make this distinction between these two behavioral responses, as well as their implications, greater throughout the manuscript and primarily in the discussion. The binary response of some individuals to evacuate vs. some to hunker down likely reflects some biological or physiological distinction that in turn influences behavior. We see some support for this in the characteristics of the tagged fish whose movement declines were influenced by sex and length. There are likely carryover effects in population structure based on who stays and who goes.

5) Distinguishing which months the authors specify as summer and fall months which likely should reference seasons based on the literature focused on fall overturn in the MAB. The authors refer to many of the storms as summer storms when they are actually occurring during the beginning of fall overturn. This is important because stratification during summer is very strong and summer storms do not typically break up stratification. Fall storms will break down stratification and is already known to be a time of significant change in the system and well documented movements of fish species;

The study period corresponds to the transitional period between summer and fall, and subsequently seasonal influences are not so easily attributed to one or the other. From a temperature point of view September is summer, but we realize this is unconventional. We have addressed this by avoiding seasonal terms in places and where we reference seasons, use conventional month intervals.

6) Explaining if there were any occurrences of fish returning after a storm event. This has been seen in some papers and would be interesting if this happened to black sea bass, especially during the earlier storms of 2017. If there is no evidence the fish returned or fish returns were not detected, that is fine but should be explained;

Yes, we did see instances of return following several days to several weeks’ time. We now report on these, but please note evacuations are analyzed by ARIMA and operationally defined as permanent evacuations. Another related aspect that in 2017, two fish returned to the same site where they were initially tagged the previous summer (Secor et al. 2019).

7) The text overall should be edited to improve clarity and readability. Some cases of this are specified in my specific comments and edits.

We are grateful for the attention paid to the readability and flow of the text. Adjustments were made throughout the document to improve its clarity, both in direct response to reviewer comments and elsewhere.

Thank you once again for the attention our manuscript. We have endeavored to match this in our revisions. For brevity, our responses to specific comments are fairly direct.

**Author response to specific comments/edits on text, tables, and figures:**

***Title***

The phrase “recurring role of storm disturbance” in the title is ambiguous. Are the authors referring to recurring storms and their impact or that there is continued evidence of impacts of storms on black sea bass?

- Line 3: Title was changed to *The recurring impact of storm disturbance on black sea	bass* (Centropristis striata) *movement behaviors in the Mid-Atlantic Bight*

***Abstract***

Line 25: Summer storms do not cause rapid destratification in the MAB. Fall storms yes, but not summer. Please revisit classification of what is considered a summer or fall storm. I have multiple comments about this in the rest of the specific comments.

- Line 24-25: “Summer” was removed in the initial description of storm events, and	events	were clarified as occurring during the late summer and early fall.

Line 28: “model ubiquitous demersal” – while black sea bass relatively stationary behavior makes them ideal for telemetry studies, it is not clear why the reader should care about depressed movements of an already stationary fish. Perhaps rephrase to emphasize “model” organism relates to their use in tagging studies but not as much on the potential impact of storms.

- Lines 27-30: Sentence was restructured to emphasize black sea bass as a model species	for the ease of which they can be tagged and also in how their sedentary, demersal life	history exposes theme regularly to storm disturbance.

Line 33: is this 8-15 black sea bass per year or over the entire 2016-2018 time frame? Please specify.

- Line 33: Clarification was included that 8-15 black sea bass were released “each year”.

Line 33: “at each of three reef sites” – this is confusing as the authors have not introduced the three reef sites yet in the abstract. I suggest rephrasing this statement.

- Line 33: “each” was removed so as to avoid confusion regarding the reef sites.

Lines 34-35: “…activity levels **and** reef departures **of black sea bass,** and fluctuations in **modeled** temperature …”

- Lines 34-36: Sentence was formatted following reviewer suggestion: “Data were	analyzed for activity levels and reef departures of black sea bass, and fluctuations in	modeled temperature, current velocity, and turbulent kinetic energy.

Lines 36-37: How do the authors reconcile differing behaviors of low activity vs. evacuation? Is this an artifact of the methodology or truly binary responses to the storm events? How do the authors know if late season departures are not associated with offshore migration?

- Lines 36-37: Methodology is discussed in greater detail further in the paper; no	adjustments made here in the abstract other than to specify that not all fish evacuated.

Lines 40-42: The conclusion of the abstract feels like a stretch. Where are anthropogenic impacts measured in this study? What are the anthropogenic impacts (i.e. fishing? Climate change? Pollution?). Perhaps a stronger conclusion is to place these results in context with climate change and the predicted increase of storm frequency and intensity along the MAB in the future.

- Lines 41-44: Conclusion was edited to emphasize how black sea bass assessments of	fishing and wind farm development impacts should be performed in the context of storm	disturbance.

***Introduction***

- General: Introduction was restructured to reflect the following: first describing the impacts of storms as a disturbance on fish; second describing the MAB and how storms may disturb the region; third, discussing potential impacts on black sea bass in the MAB and leading into hypotheses.

Line 50: “less physical structure” – This is intriguing but what might be the potential difference or concern between a region with varying degrees of physical structure?; “subject to hurricane-forcing” – this statement feels out of place because some of the other regions referenced above are not subject to hurricane forcing. Is this insinuating that we are only concerned about regions with hurricane-forcing and those with lesser storms are not as important? *This is an area where the authors could develop their ideas further. For example, what makes the MAB unique compared to these other systems is the cold pool and the ability for it to breakdown during storms. Placing this into context with the other systems referenced, especially in relation to rapid temperature changes, would provide a stronger argument to why the MAB system is important to study.

- Line 51-53: Rephrased to shift emphasis on presumption that deeper waters would be	less impacted by storms and that such systems are difficult to study.

Line 87: Rewrite as - “Thus, the literature supports that **severe** storms can disrupt demersal fish communities”. – along these lines, the examples from the literature only provide evidence of fish evacuations not reduced movement behavior…is there evidence for this in other fish species? It would be useful to provide a variety of responses of fish to storms especially because the authors found depressed movement. If this study is the first to show depressed movement, then this should be explained later in the discussion.

- Lines 63-64: Examples of severe storms driving depressed movement in reef fish	provided.

- Line 64: Inclusion of term “severe”.

Line 87-89: “Still, the concept … been fully explored” – this statement needs to be developed more. Do all studies above represent singular extreme events? Why is it important to study if storms occur each year in comparison to the other studies?

- Lines 66-71: Additional description of the unexplored potential for storms to serve as a	disturbance regime.

Line 53: “…Cape Hatteras, **North Carolina,**…”

- Line 73: “North Carolina” included to specify location of Cape Hatteras.

Line 58: “…MAB is also vulnerable to storm-driven **temperature** disturbances …”

- Line 79: “temperature” added to clarify the nature of the storm-driven disturbance.

Line 59: Remove “overlapping”.

- Line 80: “overlapping” removed.

Line 61: “…at **the** Nantucket Shoals.”

- Line 81: “the” added prior to “Nantucket Shoals”.

Line 64: References 13-16 mostly focus on fall overturn. This is fine but the seasonality of the cold pool destruction should be clearly stated. If there is more evidence of summer storms impacting the cold pool (besides this study), I would also add those and again be clear about what part of the year this is occurring.

- Lines 83-86: Terms included to directly identify the seasonality of storm-driven	overturn, beginning in the late-summer (mid-late August) and peaking in the fall	(September-October). Additional reference including documenting overturn related to a	late August storm.

Line 66: How common are nor’easters in the summer?

- Line 87: Terms “and fall storm events” added for completion; term “nor’easter”	removed.

Line 68: “summer storms” – again, this should be clarified in terms of what months “summer” refers to

- Lines 90-91: Seasonal definitions of summer and fall included.

Line 69: “rapid partial destratification” – partial vs. full destratification should be explained

- Lines 92-94: Definitions of partial and total destratification included.

Line 70: The values of temperature change are based on a hurricane event. This should be clarified as an extreme event in the system.

- Line 89: Specification of the 10 degree increase in bottom water temperature as related	to a single extreme event is provided.

Lines 70-71: Refs 18 is focused on juvenile black sea bass not adults and also gradually increased temperatures after acclimation; and refs 19-20 are on temperature tolerance not so much acclimation and very acute exposures. The impact of rapid temperature change on fish physiology is extremely important but this section needs work. Can the authors find studies of acute vs chronic exposure of increasing temperature? Studies on heat shock proteins? Also, there needs to be a link between how the physiological stress of increased temperature will affect fish behavior (i.e. movement). Is there a threshold where a fish cannot move and will not evacuate? Did any of the other papers on fish responses to storms report physiological stress? Here is also where there should be some distinguishing between behaviors of avoidance or mitigation? For example, are the fish leaving the system before the storm or compensating during and after?

- Lines 96-104: Greater specifications on behavioral and physiological descriptions of	acute and chronic heat responses across a variety of fish species provided. Also included	are results form a recent publication specific to black sea bass that shows diminished	metabolic scope as temperatures increase from 12 to 24 C.

Lines 73-75: I am intrigued about potential issues related to changes in current velocity, TKE and noise but the authors do not provide further evidence that these are stressors to fish, and as such do not provide a potential hypothesis as to how these physical effects from storms could affect fish.

- Lines 107-112: Greater specification and citation of storm generated flow disturbing	fish movement, and the potential for storm-generated noise to act as a stressor to fish	communities.

Lines 75-76: “Because several storm systems …destratification each year” – this statement is awkward, rewrite.

- Lines 112-116: Original sentence broken into two clauses for increased	clarity/readability.

Line 77: “disturbance regime” – this seems to be an important concept… the authors should explain this further and show an example of other disturbance regimes to provide evidence to why this should be unique on the MAB

- Lines 114-115: Additional description of disturbance regime as “seasonal fluctuations	in temperature and flow” included.

Line 92: “range behaviors” – this is awkward, please rephrase.

- Line 119**-**120: “range behavior” removed and replaced with “an affinity for both natural	and artificial structured habitats”.

Lines 92-94: Re-write as “…centered on artificial and natural structure [25] and are mostly sedentary and reef-associated [26-29], making them amenable to biotelemetry studies on their movement behaviors.”

- Lines 120-121: Rephrased: “which makes them an ideal candidate for biotelemetry	studies on potential shifts to their movement behaviors”.

Lines 94-97: “Black sea bass and … late October [26,27,30]” – this sentence is awkward, please rewrite this sentence

- Lines 121-124: Sentence broken into two clauses for clarity.

Line 95: “…occur **inshore** throughout the **spring**, summer and early fall months,…”

- Line 123-124: Clauses rephrased.

Lines 96-100: So here the authors explain black sea bass movements in relation to their fall migration…so are the storms analyzed in this paper not fall storms? Again, clarifying summer and fall months early on in the paper should help reduce confusion surrounding this issue.

- Lines 127-128: Arriving storms clarified as occurring in late summer and early fall.

Lines 99-100: The hypothesis from ref 17 ends the paragraph without any additional follow up from the authors. Is this also hypothesized for this paper? If not, how are the hypotheses and results of this paper placed into context with this hypothesis?

- Lines 127-129: Clause added to provide context for the hypothesis in the previous	sentence: “The potential role of late summer-fall storm disturbance in MAB to serve as a	migratory cue emphasizes the need to understand repeated storms as an ecologically-	significant disturbance regime.”

Line 101: Replace “is” with “was”

- Line 130: “is” replaced with “was”.

Line 103: Remove “More specifically” and capitalize “We..”

- Line 132: “More specifically” removed; “we” capitalized.

Lines 103-108: Are there any hypotheses related to differences in movement behavior (i.e. depressed movement vs. evacuation)?

- Lines 132-136: Left unchanged, as hypothesis-specific delineations between	evacuations and movements are described further in Methods section.

***Materials and Methods***

General comments: Section reviewed and corrected for consistent verb tense.

Line 115: Rewrite: “This project included three study reef sites located …”

- Line 144: Rewritten as: “This project included three study reefs located…”

Line 116: “…Ocean City, Maryland, **USA**, …”

- Line 145: “USA” included.

Line 118: “were exposed to” – rephrase this statement; exposed to sinuates some experimental manipulation not that these sites regularly are part of the cold pool

- Line 146: Rewritten as: “All study reefs overlapped with the presence of the cold	pool…”

Line 147: Was the tank a flow-through tank? If so, this should be mentioned. If not, were there airstones?

-Lines 173-174: Sentence “Water within the tank was partially replaced at	approximately	10-minute intervals to avoid deoxygenation.” included for clarification on	oxygen	replenishment of holding tank.

Line 151: After anesthetizing, the authors should include weighing, measuring and sexing the fish. How the authors sexed the fish is very important because they do not explain how they arrived to Unknown statuses for some of the fish in the study and this is important, especially for black sea bass.

- Lines 178-181: Additional text included to describe the weighing, measuring, and	sexing of fish prior to surgery.

Lines 153-154: “…was made cranial to the vent, and lateral to the midline.” This phrase is confusing, please rewrite.

- Line 184: Rewritten for clarity of incision location: “a 1-cm incision was made lateral to	the midline, preceding the vent”.

Lines 157-159: Did tagging the fish not help reduce some barotrauma? If the fish were fully recovered in the tanks post tagging, then how did some of them need help equalizing in the water? Were the fish vented at all? Did the fish get external tags so that if they were accidentally caught fishermen could notify the researchers?

- Lines 187-188: Statement on barotrauma observations and procedures included for	clarity: “Incisions alleviated internal pressure although barotrauma symptoms were likely	not fully abated.” We included a relevant paper on barotrauma in black sea bass and its	symptoms. We did not add additional stress by placement of external tags, but rather	tagged sublegal fish to reduce fishing takes, described earlier in the same paragraph.

Line 166: Change “Mid-Atlantic Bight” to MAB

- Line 199: “Mid-Atlantic Bight” replaced with “MAB”.

Lines 170-171: Are there citations for the ROMS ESPreSSO model?

- Line 204: Citation for ROMS provided.

Lines 177-178: The authors mention potential stress from current velocity but there is not cited evidence of this for fish nor is this idea developed in the introduction. Also what do the authors mean by “station-keeping”?

- Lines 215-218: Additional discussion of the potential effects of current velocity	included in introduction, with references included again here; “station-keeping” replaced	with “maintaining position at reef habitat home ranges”.

Line 185: “Delmarva” – this needs to be explained; most readers not from the US East Coast will not know what Delmarva means

- Lines 225-226: “Delmarva” replaced with “Delaware-Maryland-Virginia”.

Line 187: How exactly were the model results evaluated with the observed bottom water temperature? See my comments for figure SI3a-c.

- Line 227: Phrase “evaluated through comparisons with” replaced with “compared	with”.

Line 191: How and where did the authors measure peak wind speed?

- Lines 219-222: Description of how observed wind speeds were obtained.

Line 193: How was the threshold of >5m/s chosen? This seems to have filtered out less severe summer and fall storms that occur in the region. In my opinion this is fine, but the presence of only severe storms in the analysis should be explained here and in the discussion.

- Lines 234-237: Rationale for the 5 m s^-1^ wind speed cutoff provided: “The lower limit	of 5 m s^-1^ was selected to provide a conservative definition for potentially disruptive	storm activity (3 or greater on the Beaufort Wind Scale [49]). Storms above this limit	were further categorized and compared according to the Beaufort Wind Scale.”

Line 195: Change “evens” to “events”.

- Line 238: “evens” replaced with “events”.

Lines 216-217: “This approach allows discrimination … caused by storms.” – How is this interpreted to distinguish between fish leaving and coming back vs. false detections of a fish not actually leaving?

- Lines 259-269: Section of paragraph rewritten for clarity: “ARIMA intervention	analysis facilitates the identification of false evacuations, identified as single points	within the recorded time series that temporarily, but significantly, alter the behavior of	the rest of the series.” … “The analysis tested for the presence of two types of	interventions: (1) temporary shifts and (2) permanent level shifts. Permanent level shifts	(stepped declines) are indicative of fish evacuation—interventions that fundamentally	and permanently changed the remaining time series. Temporary shifts are those	interventions that altered the time series temporarily and appear as nonlinear returns to	the previous detection level (see Figure 9).”

Lines 233-235: Did the authors only run one model of 2017? If so, why did they not run models for 2016 and 2018 and remove the ANSD term? This sentence is a bit ambiguous to the reader.

- Lines 274-276: Sentence rewritten for clarity. Note that discussion of 2017 as an	optimal modeling year based on the repeated occurrence of storm events provided in	existing text.

Line 259: How were the different models constructed, considering the authors picked the best fit model based on AIC scores?

- Lines 292-294: Moved statement on AIC selection and incorporated additional criteria	for how compared models were constructed.

***Results***

Line 280: Rephrase to clarify that the storms occurred between the measured time period of June to October (because the storms did not occur in June or October)

- Line 310-312: Rephrased clarify that storms occurred during measure period of time	June-October.

Lines 282-286: The storms in September to me are early fall storms and the only true summer storm was the one at the very end of July; the August storm is also pretty late for summer. So the storms assessed are really more focused on late summer-fall storms and not “summer storms”. But if during editing this is clarified prior, then please disregard this comment.

- Lines 312-317: Text incorporated throughout Introduction and Methods sections to	clarify that storms are occurring the during the late summer and early fall.

Line 301: The relatively stable range of 12.5-16.9°C is still a significant change in bottom temperature (and significant for biology).

- Line 333-334: This observation now noted in terms of gradual rise in August; sentence	also has been simplified.

Line 305: What values are in the parentheses? Mean and SD? If so, this should be clarified.

- Line 336: Parenthesis values clarified as mean and standard deviation.

Line 306: Rewrite – “…a destratification event *at the end of* July,…”

- Line 338: Rewritten “event at the end of July”.

Line 314: “stronger storms and more moderate storms”. This seems subjective; was there a way to classify storms as “strong” vs. “moderate”?

- Line 346: Strength of storms now additionally categorized according to Beaufort Wind	Scale.

Line 322: In looking at Figure 3, the winds appear to be northeasterly not northwesterly.

- Line 356: Corrected to match patterns in Figure 3 of “northeasterly” winds.

Line 381: Replace “all” with “each”

- Line 420: “all sites” replaced with “each site”.

Line 384: Replace “lowered” with “decreased”

- Line 421: “lowered” replaced with “decreased”.

Line 385: Replace “than during” with “compared to”

- Line 422: “than during” replaced with “compared to”.

Line 385: What pairwise comparisons were significant? Is this between site? Or between years?

- No adjustment made; text indicates significance across years between given storm	events at Lines 423-425.

Lines 385-386: I am confused why the results for 2017 are only in reference to PCT10. Did the authors group together data before and after PCT10? What about the other storm systems? Again, for the Tukey contrasts, is this across sites? It would be nice to know which sites were significantly different from each other.

- Lines 424-425: Text added: “with no other significant differences detected before and	after the other storm events that year.”

Lines 394-396: Does this mean that when there are more frequent storms there is more evacuation from the shallow site compared to when there are less frequent storms?

- No adjustment made; text confirms this within Lines 452-456.

Lines 396-398: I am confused. The authors state that the instantaneous loss rates at the Middle site were the highest during all years except for 2017 and mention that this occurred when the Middle and Northern evacuation rates were similar, but really this was when the Southern site evacuation rate was higher (i.e. Middle and Northern were similar but NOT higher than Southern).

- Lines 430-432: Comparison between sites and years is not too important to the paper’s	thesis, but rather the episodic loss rates owing to storms – the point here is to quantify	baseline loss rates, which were similar across sits. This section has been reduced.

Lines 397-398: For readers unfamiliar with telemetry studies, it would be nice to put into context the difference in instantaneous loss rates across the years. There are only slight increases in rates; is this still significant? Or does it mean that there is not much interannual variability in evacuation rates all things considered?

- Line 430-432: Discussion of comparisons and role of loss rates streamlined;	calculations were also re-evaluated and corrected accordingly.

Line 437: Is this from the results of the best fit model? If the authors went through model selection it would be nice to have a clear indication of which model performed the best

- Lines 473-476: Specification that the model discussed is the best fit model (lowest AIC;	Table 5).

Line 439: So along the lines of my comment above, the best fit model still included the non-significant ANSD term?

- Yes, with mention of best fit (lowest AIC) model containing ANSD in Lines 473-476.

Lines 442-443: Remove “although it did not directly relate to storm influence on movement”. This is not quite known and it is obvious there is other biological information included in the model.

- Line 478: Removed “although it did not directly relate to storm influence on	movement”.

Line 445: If there might be an interaction between sex and length, why was this not included as an interaction? I agree that there likely is an effect of sex on length. If this is not to be used as an interaction, it would be worthwhile for the authors to provide a preliminary test showing there is or is not a relationship between sex and length.

- Relationship of sex and length likely present and detected by model, and is discussed	further in lines 484-485. Exploration of relationship relative to movement (in light of	selection bias of sample for sub-legal individuals and therefore likely females) exceeds	scope of study.

Line 449: “…entire sample.” – what do the authors mean by “sample”? I am confused by this statement

- Lines 486-487: Added “of tagged individuals”.

Line 460: Remove “the” after exhibiting.

- Line 498: “the” removed.

***Discussion***

Line 472: “…changes **in** temperature,…”

- Line 509: “in” added.

Line 483: “Early summer storms” – Not trying to beat a dead horse, but a storm occurring at the end of July does not seem “early” to me.

- Line 521: “Early summer” replaced with “Mid-summer”.

Line 494-495: It seems misleading to mention that evacuations are an extreme response yet they happened every year in the study without including information that these are also occurring during the time that we expect to see black sea bass start to move offshore.

- Lines 533-535: Sentence restructured: Evacuations are a faunal response to catastrophic	environmental change [8, 58, 59, 60], and, although their occurrence overlapped with the	fall migration period, they also occurred in each year of our study during and	immediately after storm events.

Line 504-506: Does this sentence thus indicate that the influence of Maria on water temperature did not instigate as rapid of a temperature change as the other storms? If so, this should be clarified.

- Lines 543-545: Text added: “a storm that did not instigate as rapid a change in	temperature as its predecessor.”

Lines 516-518 and 521-522: These seem to contradict each other. Do these sentences indicate that the Southern site temperature varies more in frequency than in magnitude when compared to the Middle and Northern sites? If so, please clarify.

- Lines 563-567: Text added to clarify relationship of site depth and stability: “The	shallower Southern site experienced greater short-term variability across a smaller range	of magnitude in bottom temperature, and was more prone to permanent destratification;	in contrast, the deeper Middle site experienced fluctuations across a broader range of	magnitude in bottom temperature, and did not destratify as quickly”

Lines 525-526: “The lowest rates of transmitter loss and the lowest number of evacuations occurred at the Southern site for all years” – But the authors found higher rates of instantaneous loss rates in the Southern Site in 2017…? I am confused.

- Lines 570-571: Calculations of daily transmitter loss rate were re-evaluated and	corrected. Section on comparisons and role of transmitter loss rates was reduced.

Line 530: “…relatively shallower Northern” – on the previous page the authors mention that the Northern site is deeper and similar to the Middle site. Please correct and clarify this.

- Line 575-576: Text to clarify depth relationships across sites included: “deepest Middle	vs. the slightly shallower Northern and substantially shallower”.

Lines 581-584: This paragraph is a good section to include other studies and place the results of this study into context with other fish responses to storms.

- Lines 588-590: We would prefer to retain focus on shelf systems where fish are exposed	destratification events rather than the mix of storm influences, principally in shallower	coastal systems introduced in Introduction. Only the Fabrizio study bears on such storm	impacts. We have focused the section to highlight the contrast between these two	comparable telemetry studies.

Lines 538-539: If the results from Fabrizio et al do not have the same results as the results for this study, what potential reasons can explain this? Perhaps this can shed more insight into the results found in this study (i.e. comparing the storm system of that study to the ones in this study).

- See above comments

Line 561: Refs 55-57 discuss reduced movement in relation to predatory prey experiments and indirect effects found in communities so I am confused how this can be related to reduced movement from storm impacts (these are very different mechanisms)

- We accept this point – we were using theoretical literature to support link between	movement and foraging habitat, but it was tenuous. Rather, we move directly to how	movement relates to ecological functions in black sea bass

Line 565: Ref 27 describes the biology of the South Atlantic stock of black sea bass. Please reference a paper for the northern stock of black sea bass, the species that was used in this study. Also, the September-October is towards the end of the spawning season in this region so I don’t quite know how much of an effect there would be. To me, a more important and concerning aspect is the effect of storm systems on recruitment and the ability for recently spawned gametes and larval fish to ingress during these events. Also occurring at this time is the potential for black sea bass to change sex and this should at least be addressed here as the authors found important differences between the sexes and movement behavior.

- Lines 618-619: Reference switched to Drohan et al. 2007, here and elsewhere.	Discussion of timing of individuals to change sex briefly discussed earlier in Results	(impacts beyond scope of study, as selected a biased pool towards greater proportion of	females).

Line 573: “…induce range shifts…” – what do the authors mean by this? In what way do they expect range shifts to occur (i.e. latitudinally? Cross shelf?)

- Line 627: “latitudinal” included to describe range shifts.

Line 575-588: Refer to my comment in the general comment about this paragraph.

Lines 629-648: Similar to the Abstract, this paragraph was changed to first emphasize	that black sea bass is a key species for fisheries and understanding wind energy impacts.	Greater balance is then given to both of these sectors in the context of storms and	increased storminess.

***References***

Some of the references were not inputted correctly (e.g. Ref 16, 32). Please review all references and make sure the citations are accurate.

Corrections made to references:

3. Bouchon C, Bouchon-Navaro Y, Max L. Changes in the coastal fish communities following Hurricane Hugo in Guadeloupe Island (French West Indies). Atoll Res Bull. 1994; 422: 1-19.

15. Li Y, Xue H, Bane JM. Air-sea interactions during the passage of a winter storm over the Gulf Stream: A three-dimensional coupled atmosphere-ocean model study. J Geophys Res. 2002; 107(C11): 1-13.

21. Rasmussen LL, Gawarkiewicz G, Owens WB, Lozier MS. Slope water, Gulf Stream, and seasonal influences on southern Mid-Atlantic Bight circulation during the fall-winter transition. J Geophys Res. 2005; 110(C2): 1-16.

24. Beardsley RC, Chapman DC, Brink KH, Ramp SR, Schlitz R. The Nantucket Shoals flux experiment (NSFE79). Part 1: A basic description of the current and temperature variability. J Phys Oceanogr. 1985; 15: 713-748.

25. Lentz S, Shearman K, Anderson S, Pluddemann A, Edson J. Evolution of stratification over the New England shelf during the coastal mixing and optics study, August 1996–June 1997. J Geophys Res. 2003; 108(C1): 1-14.

45. Zhang F, Li M, Ross AC, Lee SB, Zhang D. Sensitivity Analysis of Hurricane Arthur (2014) Storm Surge Forecasts to WRF Physics Parameterizations and Model Configurations. Wea Forecasting. 2017; 32(5): 1745–1764.

67. Sedberry GR, Van Dolah RF. Demersal fish assemblages associated with hard bottom habitat in the South Atlantic Bight of the U.S.A. Environ Biol Fish. 1988; 11(4): 241–258.

70. Vermaire JC, Pisaric MFJ, Thienpont JR, Mustaphi CJC, Kokelj SV, Smol JP. Arctic climate warming and sea ice declines lead to increased storm surge activity. Geophys Res Lett. 2013; 40(7): 1386–1390.

77. Öhman MC, Sigray P, Westerberg H. Offshore windmills and the effects of electromagnetic fields on fish. Ambio. 2007; 36(8): 630–633.

78. Gill AB, Huang Y, Spencer J, Gloyne-Philips I. Electromagnetic fields emitted by high voltage alternating current offshore wind power cables and interactions with marine organisms. Electromagnetics in Current and Emerging Energy Power Systems Seminar. London, UK. COWRIE. 2012.

81. Stenberg C, Støttrup JG, van Deurs M, Berg CW, Dinesen Ge, Mosegaard H, et al. Long-term effects of an offshore wind farm in the North Sea on fish communities. Mar Ecol Prog Ser. 2015; 528: 257–65.

***Tables***

Table 3: I suggest reformatting so that the respective data for each storm is easier to follow (i.e. the “Name” column looks like a string of words in a column, it took me a bit to figure out which events related to which values in the rest of the table).

Table 3: Additional lines added to clarify data across storms and rows.

***Figures***

General: There are many figures that do not have the “°” before C for temperature. Please incorporate this where it is missing.

- “°” before C added to Figures 2, S1.3.a, S1.3.b, and S1.3.c.

Figure 1: Why is the buoy indicated in the map but the data from it never used? Or if the data from it was used, then this was not clear in the manuscript. Also, there should be an inset map showing this region across the broader US East Coast for readers unfamiliar with this region.

- Buoy demarcation removed, and subset map included.

Figure 3: There should be a note about the different x-axes. I suggest instead replotting 2016 to be on the time scale as 2017 and 2018 and just begin the data for where its available.

- Replotted for consistent x-axis.

Figure 4: Similar comment to above, the x-axes should all be the same. Also the difference in the y-axes should be mentioned in the figure caption. Also the figure caption only includes a description about temperature but needs to also include it for velocity and TKE.

- Replotted for consistent x-axis; differing y-axes indicated in caption.

Figure 5: I suggest placing a box around the panel for each event that indicates when the peak winds for the storm occur. This will make it easier for the reader to view the results (which look really nice!) without flipping back and forth between the table with the dates of the storms.

- Boxed included.

Figure 6: If the authors are including three events from 2017, why not include all four? It is a bit confusing why one is not included.

- Rationale for only included first 3 storms included in caption.

Figure 8: I suggest replotting to have all graphs have the same y-axis so they can be compared across the years. Also, the date ranges are confusing for the 2017 panel. It is unclear which dates (and associated box plots) indicate before or after a storm. Also, some of the years have a lot of variance. Is there a reason for this?

- Replotted for consistent y-axis range. Figure caption altered to explicitly note grouping of	boxes.

Figure 9: The red circles and the mean behind red dashed and solid lines are not included in the figure caption. Please include this. Also, where is the data for the Northern Site 2018? I realize the model did not converge but it did not for other regions and years yet they have all the data plotted.

- Red lines corrected for consistency, and description of circles included. Northern 2018	data included (observed time series); absence of model estimates caused by inability of	model to successfully converge (described in Results).

SI1-2: Same comments as for Figure 4

- Replotted for consistent x-axis.

SI3a-c: It appears there is some model bias with FVCOM on average underestimating the observed temperatures yet this is never addressed in the manuscript. How was this dealt with? In some cases the bias was almost on the order of 5°C which is very significant for fish (e.g. Southern Site 2017).

- Bias noted and compared; further elaboration of bias beyond scope of study; note that	observed temperature was used for all modeling (not FVCOM-derived temperature).
