# Supplementary material for: The recurring impact of storm disturbance on black sea bass (Centropristis striata) movement behaviors in the Mid-Atlantic Bight
Source: PLoS One. 2020 Dec 2;15(12):e0239919. doi: 10.1371/journal.pone.0239919 (PMC7710083; doi:10.1371/journal.pone.0239919)
Supplement: Attachment — Submitted filename: 2nd Review of Wiernicki et al.docx [file pone.0239919.s012.docx]

2^nd^ review of Wiernicki et al. THE RECURRING IMPACT OF STORM DISTURBANCE ON BLACK SEA BASS (CENTROPRISTIS STRIATA) MOVEMENT BEHAVIORS IN THE MID-ATLANTIC BIGHT. Submitted to PLoS ONE. Research Article

**General Comments/summary:**

Major comments: It is clear that the authors put time, effort, and thought into their manuscript edits and this revised manuscript is much improved when compared to the first version. Especially in the introduction, methods and results, the messages are much clearer and easier to follow than before. One issue I continue to have is that the authors have chosen to still discuss wind energy in the context of storm disturbance but do not full develop the argument as to why and how we should be concerned about wind energy and storms. The major issue is that in the abstract and in the discussion the authors are vague as to whether the impacts of recurring storms will act synergistically with impacts of offshore wind development or if the information gained from studies like this will provide information as to how black sea bass will respond to the disturbance from offshore wind. I tried to provide edits throughout that may guide the authors as the mentions of wind energy still read as a tangential issue to the concerns in this study. The discussion still needs some considerable editing (see in specific comments). Finally, especially in the newly revised sections, there are still areas that need general editing, mostly for grammar. I tried to point this out where it occurred but I suggest all authors re-read and edit the manuscript before resubmission.

**Specific comments/edits – Text, Tables, Figures:**

***Abstract***

Specific (bold = insert):

Line 24: I suggest adding some statement after Middle Atlantic Bight such as “along the U.S. Northeast” or “in the Northwest Atlantic” so that readers outside of the U.S will know where this study takes place. Finally, remove the abbreviation because the MAB abbreviation is used only one other time (Line 28).

Line 40: “…, effect**s**.”

Line 42: “US”. The authors have not defined the abbreviation of “US”, which may be obvious to some, but should be avoided for readers outside of the United States.

Lines 42-44: This sentence is vague and not well written, so I am unsure what message it is trying to convey. What do the authors mean “in context of storm as a recurring natural disturbance”? This is where a distinction between understanding natural disturbances to aid in predictions of future anthropogenic disturbances vs. concerns with confounding effects of storms and wind energy should be made.

***Introduction***

General: Great improvement. The introduction reads smoothly and provides convincing evidence for why studies like the one here is needed. Only minor edits for this section.

Specific (bold = insert):

Line 64: “…tighter coupling of fish to structured **habitat**.”

Line 64-65: Remove “Thus, the literature supports that severe storms can be disruptive events to fish communities” and insert the sentence beginning with, “**Thus**, the vast majority of storm impacts on marine communities…” and remove “Still,” and replace with “and” to combine these sentences.

Lines 105-106: Remove “, including black sea bass” as black sea bass as a focal study species has not been introduced yet.

Line 114: Perhaps at the end of this sentence the authors can include some information about offshore wind development and the impacts it can have on fish. Here the authors can introduce for the first time whether they are discussing offshore wind as an additive effect to storms or that storm disturbance studies can be used to understand the impacts of offshore wind development.

Line 122-123: “…characteristically occupy shelf natural …to late October”. This phrase is redundant from the one above in Line 120. Consider editing.

***Materials & Methods***

General: Overall, great improvements to the methods section. This section is much clearer and I appreciated the addition of the Beaufort scale to help compare the different storms. Only minor edits here.

Specific (bold = insert):

Line 179-180: The information about how to sex black sea bass needs to be cited. Also it is unclear if the authors are visually inspecting after they make an incision for the tag, if they are sexing via a cannula, or if they are merely using abdominal pressure to expel milt or eggs (especially as they seemed to have issues with sexing a good amount of fish and I am curious as to why). Finally, the authors should make a reference that their collection period overlaps with around the peak spawning time.

Lines 262: Remove “those” and add “data” before “points”

Lines 270-271: “This analysis was **performed** in R, …”

Lines 276-277: “The predictors were tested for **their** influence on **the response variable,** daily average movement**, and** included daily average TKE, …”

***Results***

General: Great job editing the results, no comments here.

***Discussion***

General: Overall, this section needs the most attention. It is hard to follow the train of thought throughout the discussion, which leads to some sections reading as redundant to previous ones and ultimately dilutes the important messages. For example, the authors discuss evacuations in two different paragraphs that are separated by 2 other paragraphs that discuss physical oceanography and stable vs. unstable temperatures and fish movements. The authors also discuss limitations to their study and then continue to discuss their results in specific context to black sea bass life history. The final paragraph discussing the results in context to fisheries and offshore wind development requires considerable editing if the authors wish to include this information in the manuscript. Specific comments below aim to address these concerns.

Specific (bold = insert):

Line 509 and 520: These paragraphs are slightly redundant; consider combining.

Line 535-539: “Biotelemetry detections … permanent evacuations.” Either remove this information or add it to the limitations paragraph.

Line 539: Remove “where” and replace with “when”

Line 545-547: “This suggestions … in movement behavior.” This is an important result and is buried in text within the paragraph. I suggest moving this information to the beginning of the paragraph to highlight the authors important findings.

Line 551: Remove “of” and replace with “to”

Line 564 and Line 566: Remove “magnitude in” and make temperature plural

Line 568: “**Our f**indings …”

Line 577: This paragraph needs editing. The impacts on population dynamics and what the authors mean by population dynamics is vague. Perhaps the other paragraph later in the discussion about black sea bass reproduction can be incorporated into this paragraph.

Line 578: “..are distinct”. Distinct responses? Behaviors?

Lines 582-583: Edit to “late-summer fall, may impact habitat use…”

Line 587: Change “don’t” to “do not”

Lines 588-589: This topic sentence needs editing. What do the authors mean by inferences?

Line 597: Edit to “…relate to study design include assumptions…”

Line 615: This paragraph either needs to be developed more or incorporated with another paragraph (see above).

Line 619: The Drohan et al 2007 citation is wrong; the studies that show late season spawning are included within the Drohan paper

Line 620: The potential impacts on black sea bass spawning are a stretch and need to be clarified that this late season spawning is rare and the typical spawning season is earlier in the summer and would not necessarily be affected (but maybe during years like 2017). The papers within the Drohan paper that claim late season spawning are old papers and estimate black sea bass spawning through egg and larval black sea bass surveys.

Line 627: It is unclear why storms would lead to latitudinal range shifts. Either develop this concept more or remove it.

Line 631: “erected” is an awkward word choice

Line 632: I suggest reordering fishing and wind farm impacts to reflect the order in which these are discussed in the paragraph

Line 631-636: Here is where the authors need to clarify because section is vague as to whether lessons from this study can aid in predictions of construction impacts or if we need to think about storms and construction impacts together. If I understand correctly, wind farm development and increasing storm impacts could lead to lower abundances of fish in those regions and this needs to be considered in the context of fisheries.

Line 633: Edit to “…will occur **during** periods and **in** regions…”

Line 637-639: “Each of these stresses can…construction alone.” How? Are there examples? Is this speculation? Obscuring and enhancing impacts are opposite effects, so is there a potential storms interacting with wind farms is beneficial?

Line 640: “…catchability in particular.” The authors after here transition to anecdotal information about how storms can reduce catchability yet the above sentence is in reference to pile driving. So now it would appear the authors are using the impacts of storms as a way to understand the impacts of offshore wind development, where before they were discussing the effects acting in concert with one another.
