# Supplementary material for: The recurring impact of storm disturbance on black sea bass (Centropristis striata) movement behaviors in the Mid-Atlantic Bight
Source: PLoS One. 2020 Dec 2;15(12):e0239919. doi: 10.1371/journal.pone.0239919 (PMC7710083; doi:10.1371/journal.pone.0239919)
Supplement: Attachment — Submitted filename: Response to Reviewers.docx [file pone.0239919.s013.docx]

Dear Dr. Magar:

Thank you for your very thorough second review. We greatly appreciate your commitment to improving this manuscript. Again, we have addressed all general and specific comments below, referencing line numbers for more substantive changes in the clean/marked copy.

Sincerely Yours,

Caroline Wiernicki, NOAA Knauss Fellow

cc:	M O’Brien

F Zhang

V Lyubchich

M Li

D Secor

Review of Wiernicki et al. THE RECURRING IMPACT OF STORM DISTURBANCE ON BLACK SEA BASS (CENTROPRISTIS STRIATA) MOVEMENT BEHAVIORS IN THE MID-ATLANTIC BIGHT. Submitted to PLoS ONE. Research Article

**General Comments/summary:**

Major comments: It is clear that the authors put time, effort, and thought into their manuscript edits and this revised manuscript is much improved when compared to the first version. Especially in the introduction, methods and results, the messages are much clearer and easier to follow than before. One issue I continue to have is that the authors have chosen to still discuss wind energy in the context of storm disturbance but do not full develop the argument as to why and how we should be concerned about wind energy and storms. The major issue is that in the abstract and in the discussion the authors are vague as to whether the impacts of recurring storms will act synergistically with impacts of offshore wind development or if the information gained from studies like this will provide information as to how black sea bass will respond to the disturbance from offshore wind. I tried to provide edits throughout that may guide the authors as the mentions of wind energy still read as a tangential issue to the concerns in this study. The discussion still needs some considerable editing (see in specific comments). Finally, especially in the newly revised sections, there are still areas that need general editing, mostly for grammar. I tried to point this out where it occurred but I suggest all authors re-read and edit the manuscript before resubmission.

Thank you for your careful review. Regarding major comments on the argument on wind energy concerns: we have followed your guidance and incorporated more specific dialogue linking uncertainties from wind farm construction disturbance relative to storm disturbance, and how the interaction may impact local abundance for Mid-Atlantic fisheries. Regarding concerns over general edits to grammar and flow, all authors have re-read the manuscript and provided edits.

Thank you once again for the attention to our manuscript. We have endeavored to match this in our revisions. For brevity, our responses to specific comments are fairly direct.

**Author response to specific comments/edits on text, tables, and figures:**

***Abstract***

Line 24: I suggest adding some statement after Middle Atlantic Bight such as “along the U.S. Northeast” or “in the Northwest Atlantic” so that readers outside of the U.S will know where this study takes place. Finally, remove the abbreviation because the MAB abbreviation is used only one other time (Line 28).

- Lines 24-25: Phase “in the Northwest Atlantic” added to clarify broader geographic area	of interest.

- Lines 28-29: Abbreviation “MAB” replaced with “Middle Atlantic Bight”.

Line 40: “…, effect**s**.”

- Line 41: “effect” pluralized.

Line 42: “US”. The authors have not defined the abbreviation of “US”, which may be obvious to some, but should be avoided for readers outside of the United States.

- Line 43: “US” replaced with “United States”.

Lines 42-44: This sentence is vague and not well written, so I am unsure what message it is trying to convey. What do the authors mean “in context of storm as a recurring natural disturbance”? This is where a distinction between understanding natural disturbances to aid in predictions of future anthropogenic disturbances vs. concerns with confounding effects of storms and wind energy should be made.

- Lines 43-45: Sentence rephrased, “Their availability to fisheries surveys and sensitivity	to wind turbine impacts will be biased during periods of high storm activity, which is	likely to increase with regional climate change.”

Line 64: “…tighter coupling of fish to structured **habitat**.”

- Line 73: Term “habitat” added.

Line 64-65: Remove “Thus, the literature supports that severe storms can be disruptive events to fish communities” and insert the sentence beginning with, “**Thus**, the vast majority of storm impacts on marine communities…” and remove “Still,” and replace with “and” to combine these sentences.

- Lines 73-75: Sentence rephrased: “Marine community responses to even single storm	events represents challenging field science, perhaps contributing to a lack of studies on	multiple storm events as a recurring source of natural disturbance.”

Lines 105-106: Remove “, including black sea bass” as black sea bass as a focal study species has not been introduced yet.

- Line 121: Removed “, including black sea bass”.

Line 114: Perhaps at the end of this sentence the authors can include some information about offshore wind development and the impacts it can have on fish. Here the authors can introduce for the first time whether they are discussing offshore wind as an additive effect to storms or that storm disturbance studies can be used to understand the impacts of offshore wind development.

- Line 146-177-131: Sentences added, “Comprehensive understanding of this natural	disturbance regime shaped by storms is both timely and relevant to the MAB, as the region	is currently undergoing evaluation by industry and policy stakeholders for offshore wind	energy development [43]. Black sea bass is a model species for understanding wind energy	impacts because they are ubiquitous and commercially important within the > 7 10^3^ km^2^ of	leased US Federal waters (<https://www.boem.gov/renewable-energy/state-activities>).. To	best utilize this and similar demersal species in understanding both negative and positive	impacts of offshore wind energy development, baseline information is needed on storm	impacts to black sea bass. Storm effects on fish behavior are a pervasive recurrent natural	disturbance in the MAB, which if not understood and accounted for, will likely bias wind	turbine impact studies.”

Line 122-123: “…characteristically occupy shelf natural …to late October”. This phrase is redundant from the one above in Line 120. Consider editing.

- Line 138: Sentence rephrased, “…nearshore shelf habitats…”

***Materials and Methods***

Line 179-180: The information about how to sex black sea bass needs to be cited. Also it is unclear if the authors are visually inspecting after they make an incision for the tag, if they are sexing via a cannula, or if they are merely using abdominal pressure to expel milt or eggs (especially as they seemed to have issues with sexing a good amount of fish and I am curious as to why). Finally, the authors should make a reference that their collection period overlaps with around the peak spawning time.

Line 234-236: Additional text added to clarify procedure for determining sex, “Sex was	determined by the visual inspection and identification of gonads, which were visible	during surgery through the incision (see below) [44].”

- Line 236: Citation added for sex determination methodology.

Lines 262: Remove “those” and add “data” before “points”

- Line 333: Removed “those” and added term “data”.

Lines 270-271: “This analysis was **performed** in R, …”

- Line 343: Removed “carried out” and replaced with “performed”.

Lines 276-277: “The predictors were tested for **their** influence on **the response variable,** daily average movement**, and** included daily average TKE, …”

- Lines 348-349: Terms added for clarification: “The predictors were tested for their	influence on the response variable, daily average movement index, and included daily	average TKE, observed…”

***Discussion***

Line 509 and 520: These paragraphs are slightly redundant; consider combining.

- Lines 589-599: Paragraphs combined; sentence removed as it was redundant, “Results	also indicated that multiple storm events in a given year may precondition the impacts of	subsequent storms on water column stratification, though we failed to detect a cumulative	impact of repeated storms on black sea bass movement.”

Line 535-539: “Biotelemetry detections … permanent evacuations.” Either remove this information or add it to the limitations paragraph.

- Line 624: Sentence removed, “Biotelemetry detections can be biased low during storm	events when ambient noise interferes with detection of transmitted signals; we conducted	analyses robust to this source of bias through an ARIMA intervention analysis, and	observed that, in all years, late-season storms were associated with permanent evacuations.”

Line 539: Remove “where” and replace with “when”

- Line 624: Replaced “where” with when”.

Line 545-547: “This suggestions … in movement behavior.” This is an important result and is buried in text within the paragraph. I suggest moving this information to the beginning of the paragraph to highlight the authors important findings.

- Lines 622-624: Information moved earlier in paragraph, “Furthermore, analysis of	storm-driven environmental variables during the multiple-storm year, 2017, indicated	storm-driven destratification was the primary catalyst for evacuations.”

Line 551: Remove “of” and replace with “to”.

- Line 648: Replaced “of” with “to”.

Line 564 and Line 566: Remove “magnitude in” and make temperature plural

- Lines 661-662: Removed “magnitude in” and made “temperature” plural.

Line 568: “**Our f**indings …”

- Line 668: “Our” added.

Line 577: This paragraph needs editing. The impacts on population dynamics and what the authors mean by population dynamics is vague. Perhaps the other paragraph later in the discussion about black sea bass reproduction can be incorporated into this paragraph.

- Line 677-688: Sentences rephrased/added, “The two behavioral responses measured in	this study—evacuations or decreased movement in the face of acute disturbance to	habitat	conditions—are distinct modes, which may have carryover effects to feeding,	reproduction, and predator evasion. Black sea bass occupy small home ranges on	structured habitats (0.14-7.4 km^2^) [40], where they feed on reef and adjacent seabed prey	items [38, 63, 64]. Reproduction has been observed to occur primarily during June-	September [38] and occurs frequently with spawning intervals for females estimated to be	2.7 to 4.6 days [65] . Thus late-summer and early-fall storms, which caused depressed	movements away from structure likely interfere with feeding and courtship, which occurs	in regions adjacent to the reef. The alternate behavior, evacuation, likely disrupts mating	systems and feeding territories and incurs greater predation risk. Additional research is	called for on changed feeding and reproductive states before and after storms, the fate of	evacuees, and whether evacuations contribute to the greater fall seasonal migration to	deeper shelf habitats.”

Line 578: “..are distinct”. Distinct responses? Behaviors?

- Line 68: Term “modes” added.

Lines 582-583: Edit to “late-summer fall, may impact habitat use…”

- Lines 677-688: Original phrase removed.

Line 587: Change “don’t” to “do not”.

- Line 677-688: Original phrase removed

Lines 588-589: This topic sentence needs editing. What do the authors mean by inferences?

- Line 749: Phrase included for clarification, “…on movement behaviors…”

Line 597: Edit to “…relate to study design include assumptions…”

- Line 758: Sentence edited, “Key limitations to our findings related to study design	include…”

Line 615: This paragraph either needs to be developed more or incorporated with another paragraph (see above).

- Lines 677-688: Paragraph edited and features incorporated into earlier discussion on	differential impacts to population dynamics.

Line 619: The Drohan et al 2007 citation is wrong; the studies that show late season spawning are included within the Drohan paper.

-Line 682: Drohan citation switched with Berrien and Sibunka 1999.

Line 620: The potential impacts on black sea bass spawning are a stretch and need to be clarified that this late season spawning is rare and the typical spawning season is earlier in the summer and would not necessarily be affected (but maybe during years like 2017). The papers within the Drohan paper that claim late season spawning are old papers and estimate black sea bass spawning through egg and larval black sea bass surveys.

- Line 681-682: Additional information on spawning included, particularly that black sea	bass are frequent spawners so events that cause several d periods of depressed activity	likely impact courtship and reproduction, “Reproduction has been observed to occur	primarily during June-September [38] and occurs frequently with spawning intervals for	females estimated to be 2.7 to 4.6 days [65] .”

Line 627: It is unclear why storms would lead to latitudinal range shifts. Either develop this concept more or remove it.

- Line 794: “latitudinal range shifts” removed.

Line 631: “erected” is an awkward word choice.

- Line 798: Replaced “erected” with “established”.

Line 632: I suggest reordering fishing and wind farm impacts to reflect the order in which these are discussed in the paragraph

- Line 799: Order of wind farm and fishing impacts switched to reflect discussion in	paragraph.

Line 631-636: Here is where the authors need to clarify because section is vague as to whether lessons from this study can aid in predictions of construction impacts or if we need to think about storms and construction impacts together. If I understand correctly, wind farm development and increasing storm impacts could lead to lower abundances of fish in those regions and this needs to be considered in the context of fisheries.

- Lines 866-867: Sentence added, “Furthermore, collective impacts from wind farm	construction coupled with impacts from natural storm disturbance could lead to altered	fish abundances in regions along the cold pool front.”

Line 633: Edit to “…will occur **during** periods and **in** regions…”

- Line 800: Terms “during” and “in” added accordingly.

Line 637-639: “Each of these stresses can…construction alone.” How? Are there examples? Is this speculation? Obscuring and enhancing impacts are opposite effects, so is there a potential storms interacting with wind farms is beneficial?

- Lines 858-862: Additional sentence added to paragraph for clarification, ““Refuge-	seeking behaviors associated with pile-driving or vessel noise may be similar in kind to	depressed movements associated with storms. Storm-induced evacuations could obscure	departures associated with wind turbine construction. Similarly, reduced catchability	could be erroneously associated with wind turbine impacts following a period of high	storm activity.”

Line 640: “…catchability in particular.” The authors after here transition to anecdotal information about how storms can reduce catchability yet the above sentence is in reference to pile driving. So now it would appear the authors are using the impacts of storms as a way to understand the impacts of offshore wind development, where before they were discussing the effects acting in concert with one another.

- Lines 863-873: Thanks, we have altered text so reduced catchability is not linked to pile	driving but rather use the last paragraph to speculate how wind farm and storm impacts	could jointly impact distributional changes. “The nature of the potential interaction of	natural storm disturbance with wind farm construction disturbance is not well known, and	future research on whether this interaction is beneficial or detrimental to fish abundance	in affected regions is critical for future management. Furthermore, collective impacts	from wind farm construction coupled with impacts from natural storm disturbance could	lead to altered fish abundances in regions along the cold pool front. Anecdotal reports	from charter fishers suggest greatly reduced catch rates following major storms (D.	Zemeckis, Rutgers University, pers. comm.). Storms may also catalyze fall departures to	deeper shelf environments shifting fisheries and influencing their accessibility to bottom	trawl surveys [85]. The need for storm disturbance to be incorporated in baseline and	impact monitoring is heightened by the prediction of higher occurrence of higher	intensity storms related to climate change.”
